# Supplementary material for: Structural framework for the assembly of the human tRNA ligase complex
Source: Nat Commun. 2026 Sep 17;17:9883. doi: 10.1038/s41467-026-77450-y (PMC13586351; doi:10.1038/s41467-026-77450-y)
Supplement: Supplementary file 1 [file 41467_2026_77450_MOESM1_ESM.pdf]

Supplementary Information for: Structural framework for the assembly of the human tRNA ligase complex

Moritz M Pfleiderer[1], Moritz Kleinwächter[2,3], Ajse S. Nievergelt[1], Franziska M Boneberg[1], Alena Kroupova[1], Javier Martinez[2] & Martin Jinek[1,4]

[1] Department of Biochemistry, University of Zurich, Winterthurerstrase 190, 8057 Zurich, Switzerland
[2] Max Perutz Labs, Max Perutz Labs, Medical University of Vienna, Vienna BioCenter (VBC), Dr. Bohr-Gasse 9/2, 1030 Vienna, Austria
[3]Vienna Biocenter PhD Program, a Doctoral School of the University of Vienna and the Medical University of Vienna, 1030 Vienna, Austria.

[4] Lead contact

Correspondence: jinek@bioc.uzh.ch

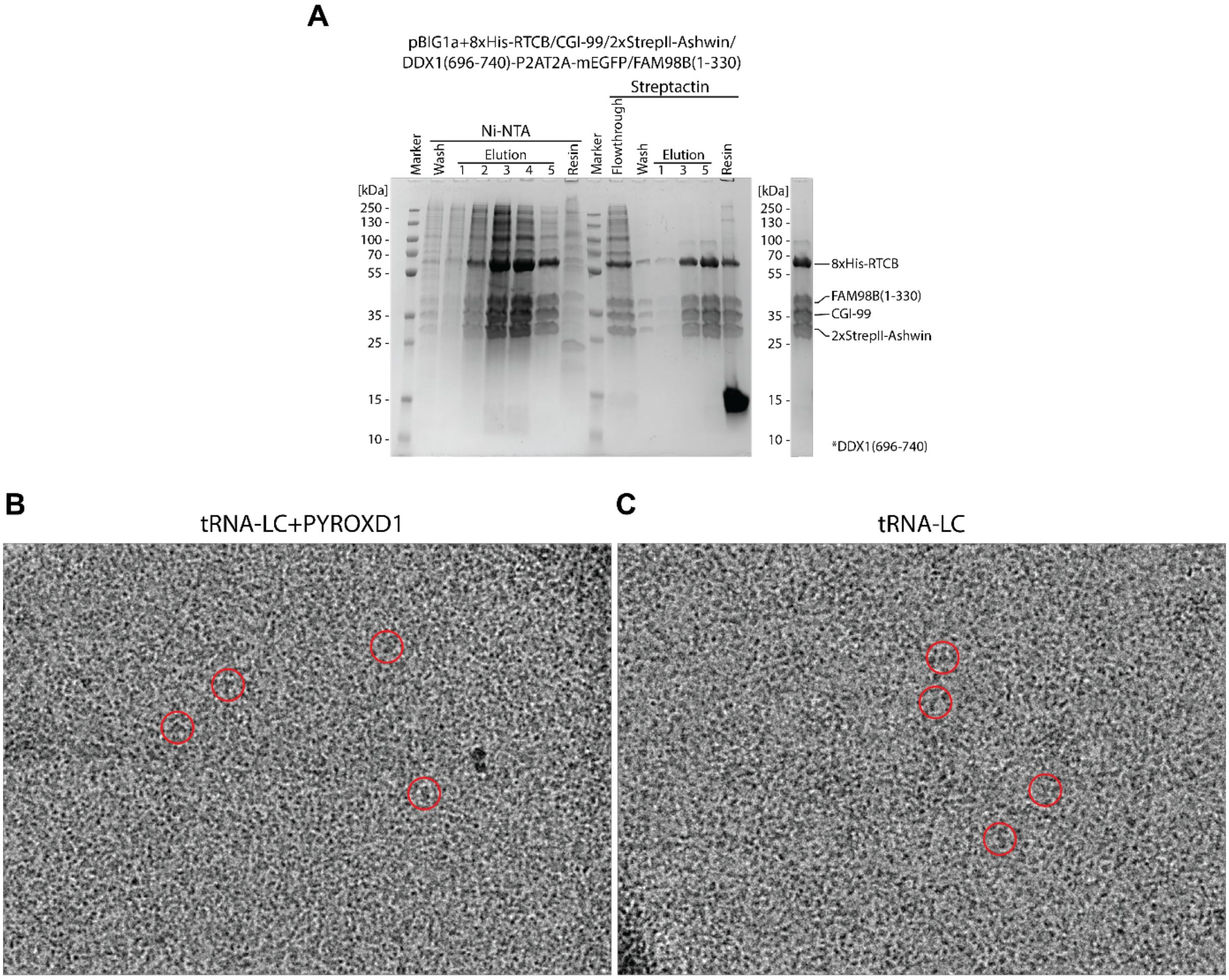

Figure S1 | Recombinant expression and purification of tRNA-LC.

(**A**) Two-step purification of the tRNA-LC expressed in insect cells. In the first purification step recombinant tRNA-LC was purified by Ni-NTA affinity chromatography. The second purification step used Streptactin-affinity chromatography and desthiobiotin elution. (**B,C**) Representative micrographs of vitrified tRNA-LC (**B**) and tRNALC+PYROXD1 (**C**). Example particles are circled in red.

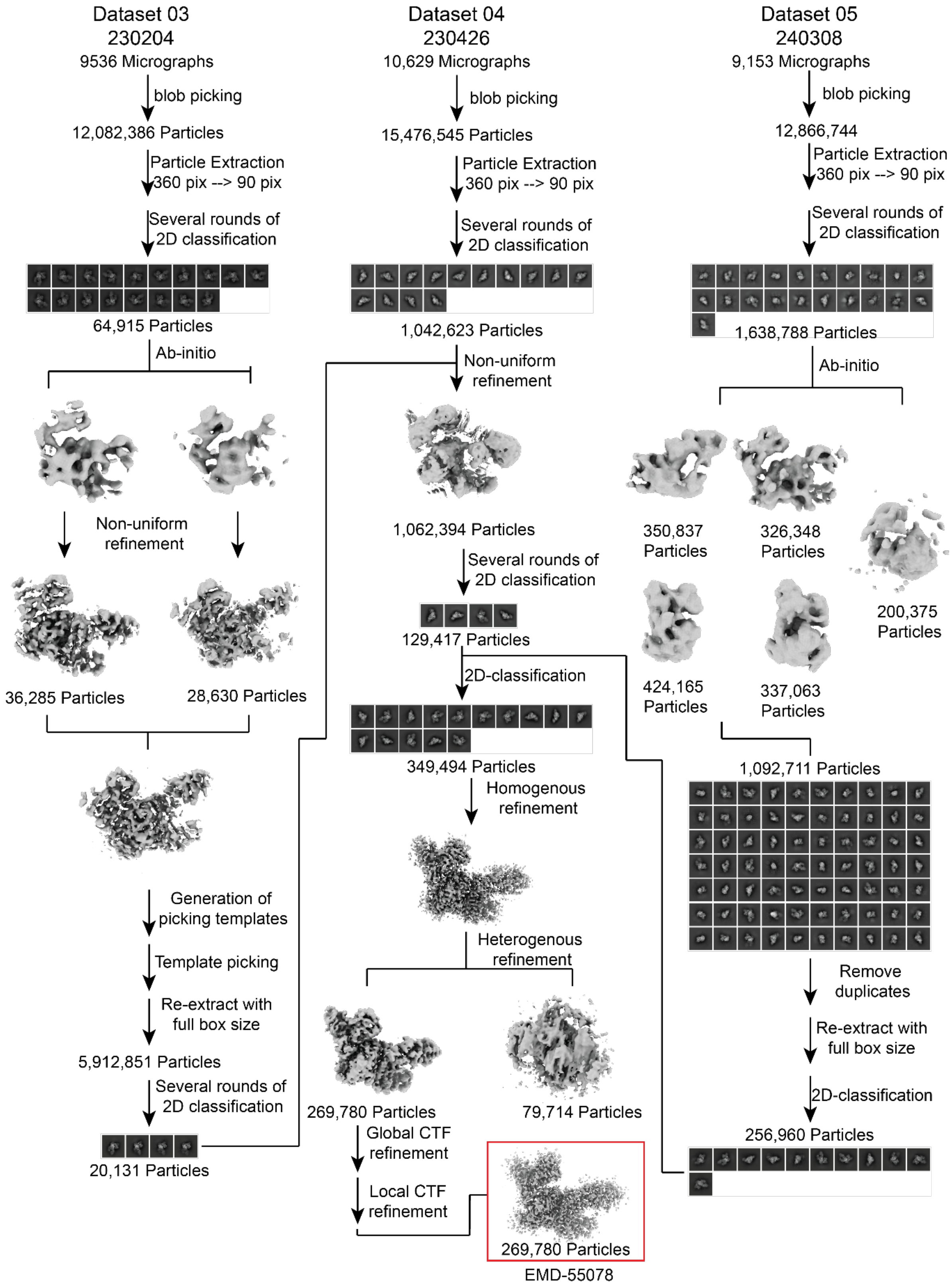

Figure S2 | Cryo-EM data processing workflow for tRNA-LC complexed with PYROXD1

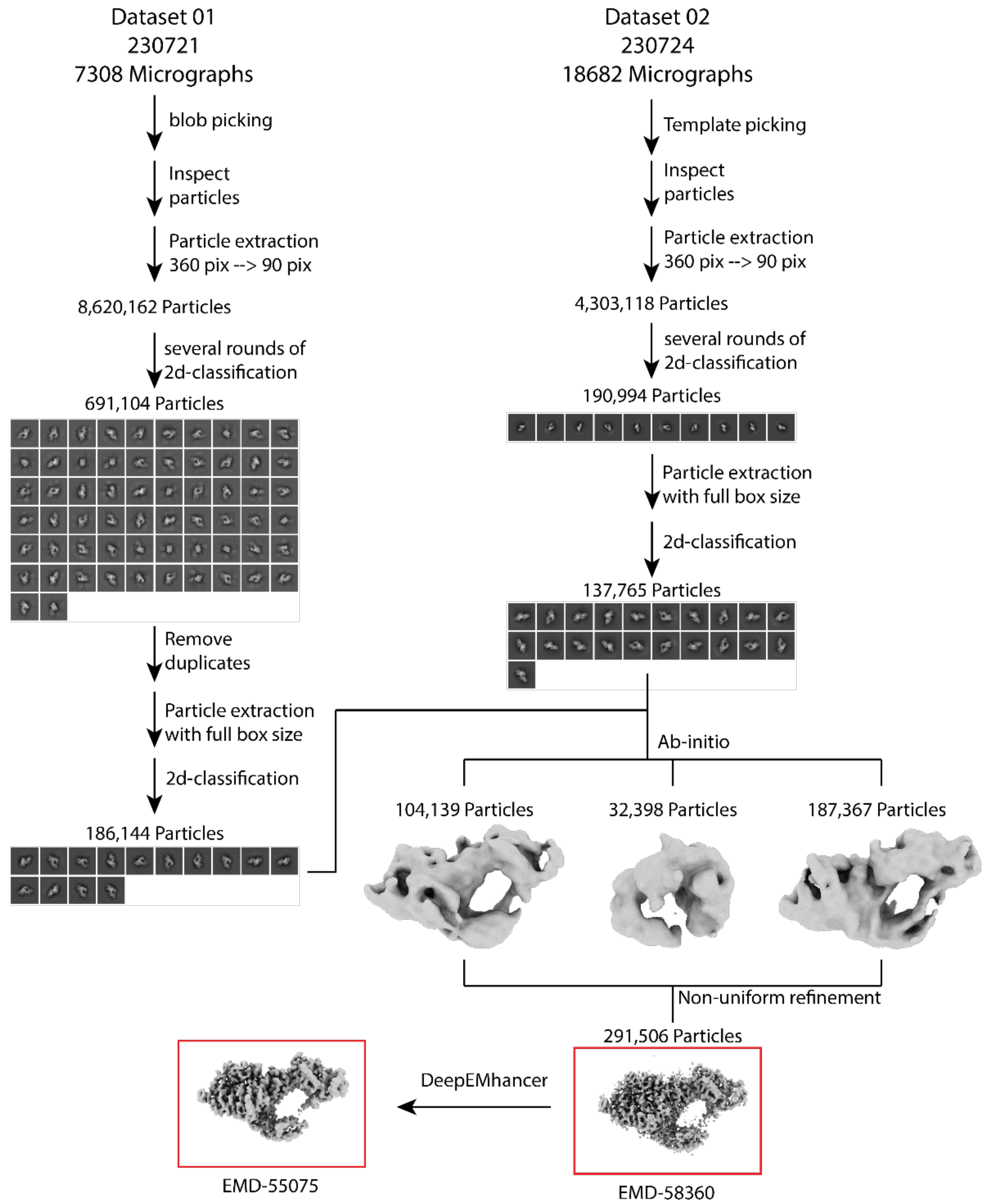

Figure S3 | Cryo-EM data processing workflow for heteropentameric tRNA-LC.

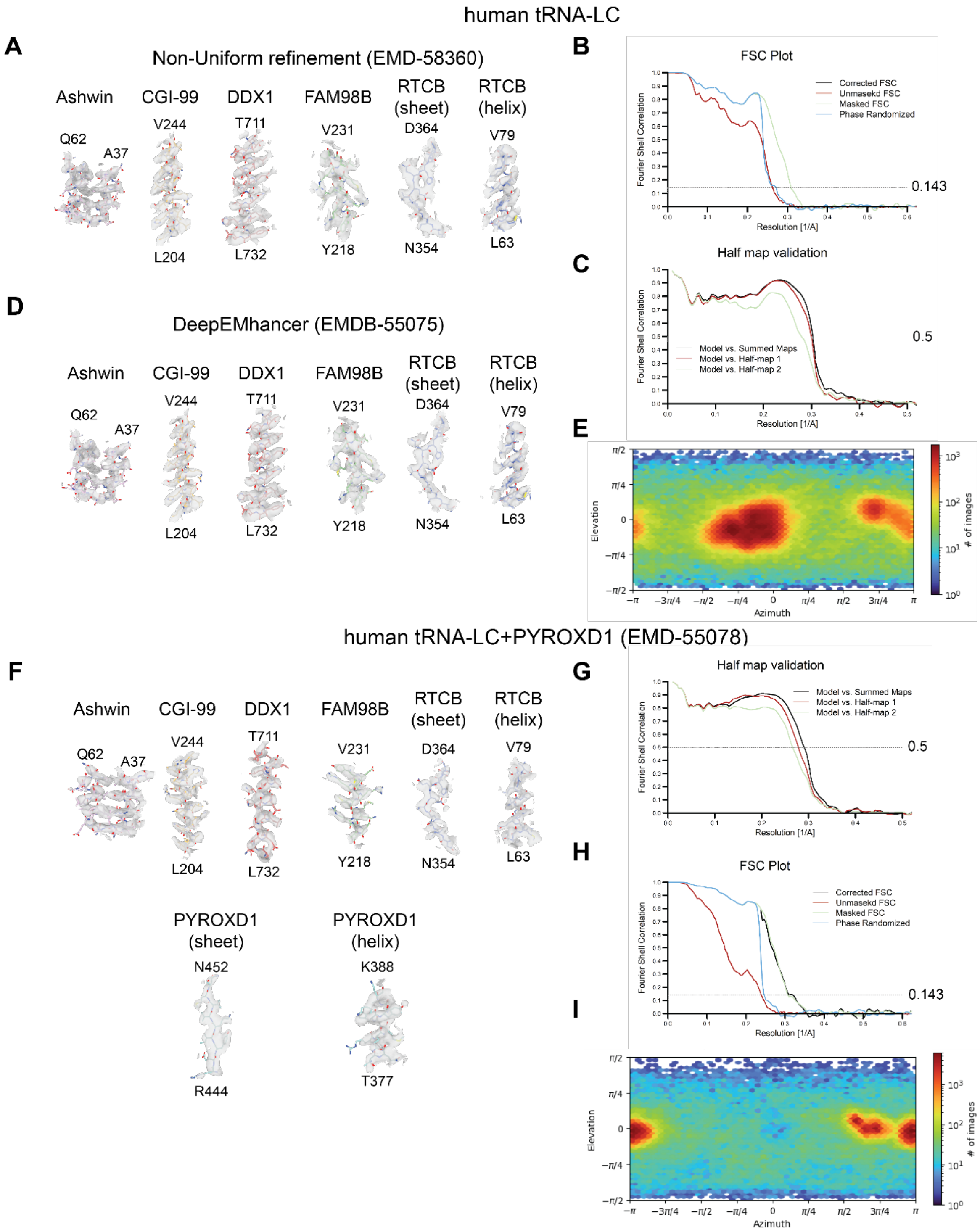

Figure S4 | Cryo-EM model and map quality

(**A**) Representative helices and β-sheets modeled in corresponding cryo-EM densities for the pentameric tRNA-LC. (**B**) FSC-plot for the tRNA-LC density. (**C**) Model validation plots for the tRNA-LC. (**D**) Representative helices and β-sheets modelled in corresponding cryo-EM densities for the tRNA-LC after processing with DeepEMhancer. (**E**) Orientation distribution plot for the tRNA-LC density. (**F**) Representative helices and β-sheets modelled in corresponding cryo-EM densities for tRNA-LC in complex with PYROXD1. (**G**) FSC-plot plot for the density of tRNA-LC in complex with PYROXD1. (**H**) Model validation plots for tRNA-LC in complex with PYROXD1. (**I**) Orientation distribution plot for the density of the tRNA-LC in complex with PYROXD1.

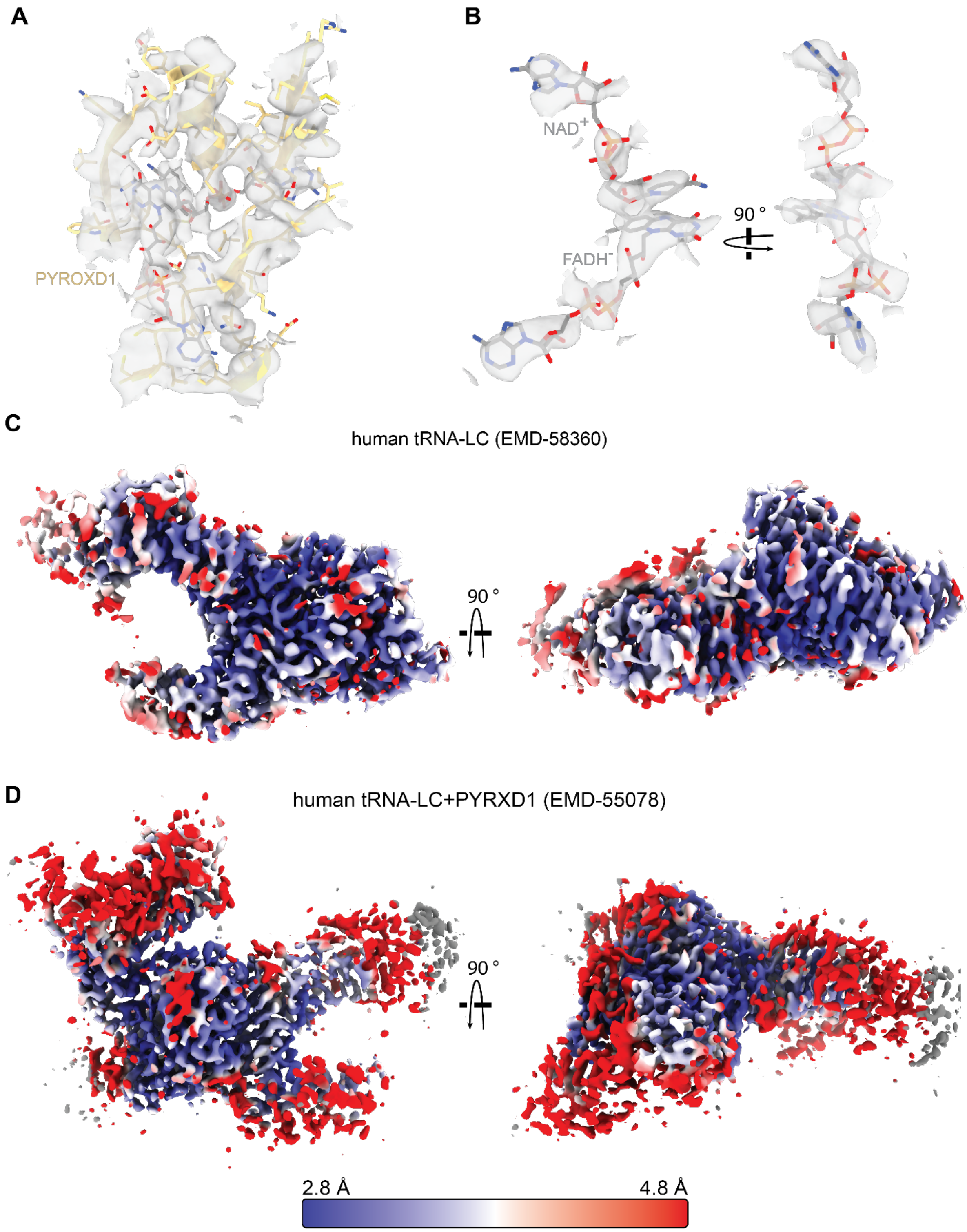

Figure S5 | Cryo-EM map quality and local resolution

(**A**) FADH- and NAD+ ligands of the PYROXD1 bound tRNA-LC modeled in corresponding cryo-EM densities with surrounding residues. (**B**) Close-up on the FADH- and NAD+ ligands of the PYROXD1 bound tRNA-LC. (**C,D**) Local resolution coloring of the human tRNA-LC (**C**) and PYROXD1 bound tRNA-LC (**D**).

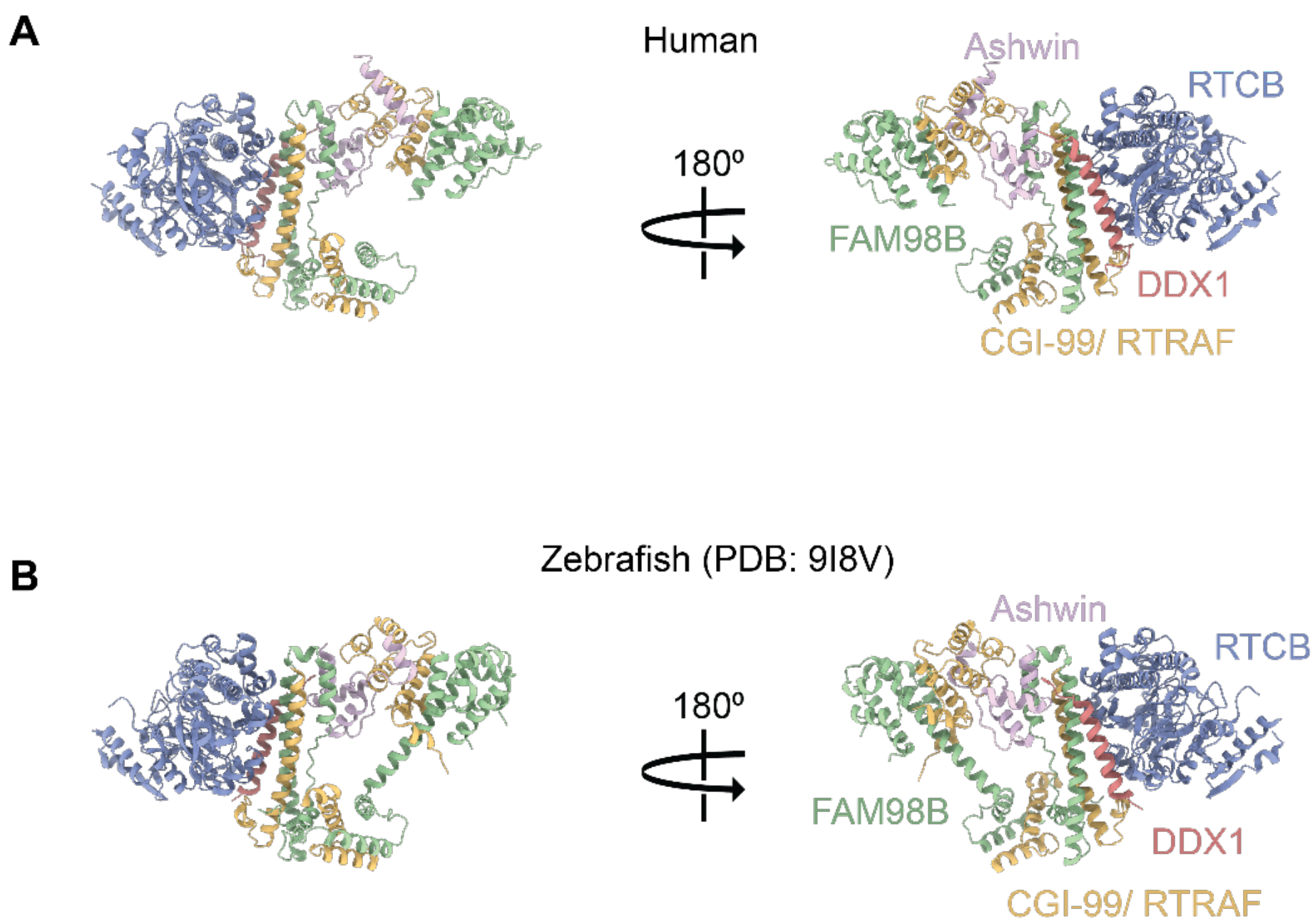

Figure S6 | Comparison of human and zebrafish tRNA-LC structures

(**A**) Human tRNA ligase complex (tRNA-LC) structure presented in this study. (**B**) Structure of zebrafish tRNA-LC (PDB: 9I8V). Homologous subunits are shown in the same colors.

A

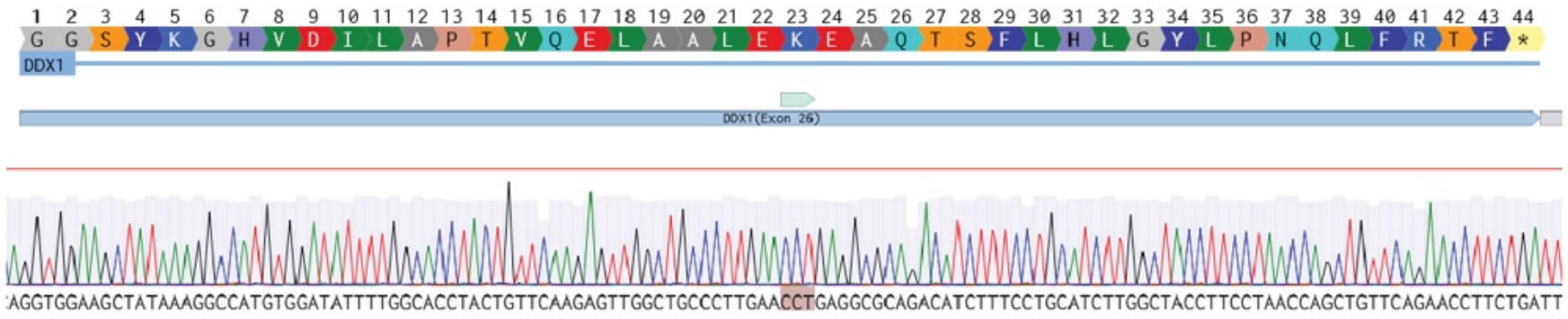

B

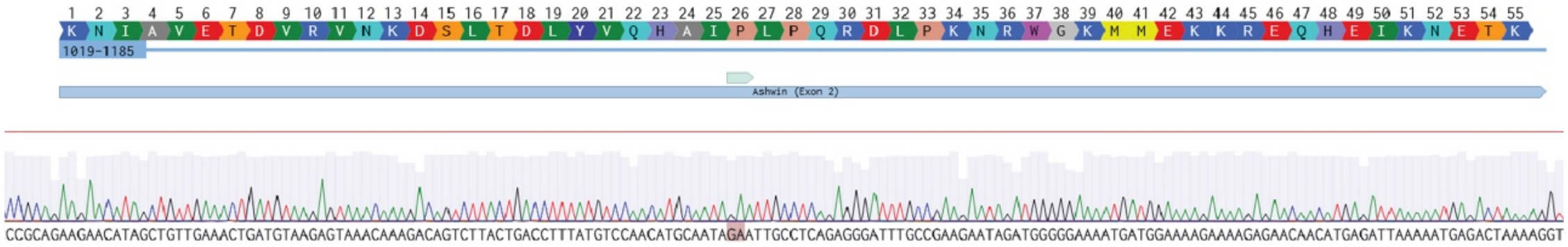

Figure S7 | Validation of DDX1 and ASW genome-edited cell lines

(**A-B**) Sanger sequencing results confirming the presence of the DDX1 K720P (**A**) and ASW P59E (**B**) mutations in genome-edited HEK293T cells.

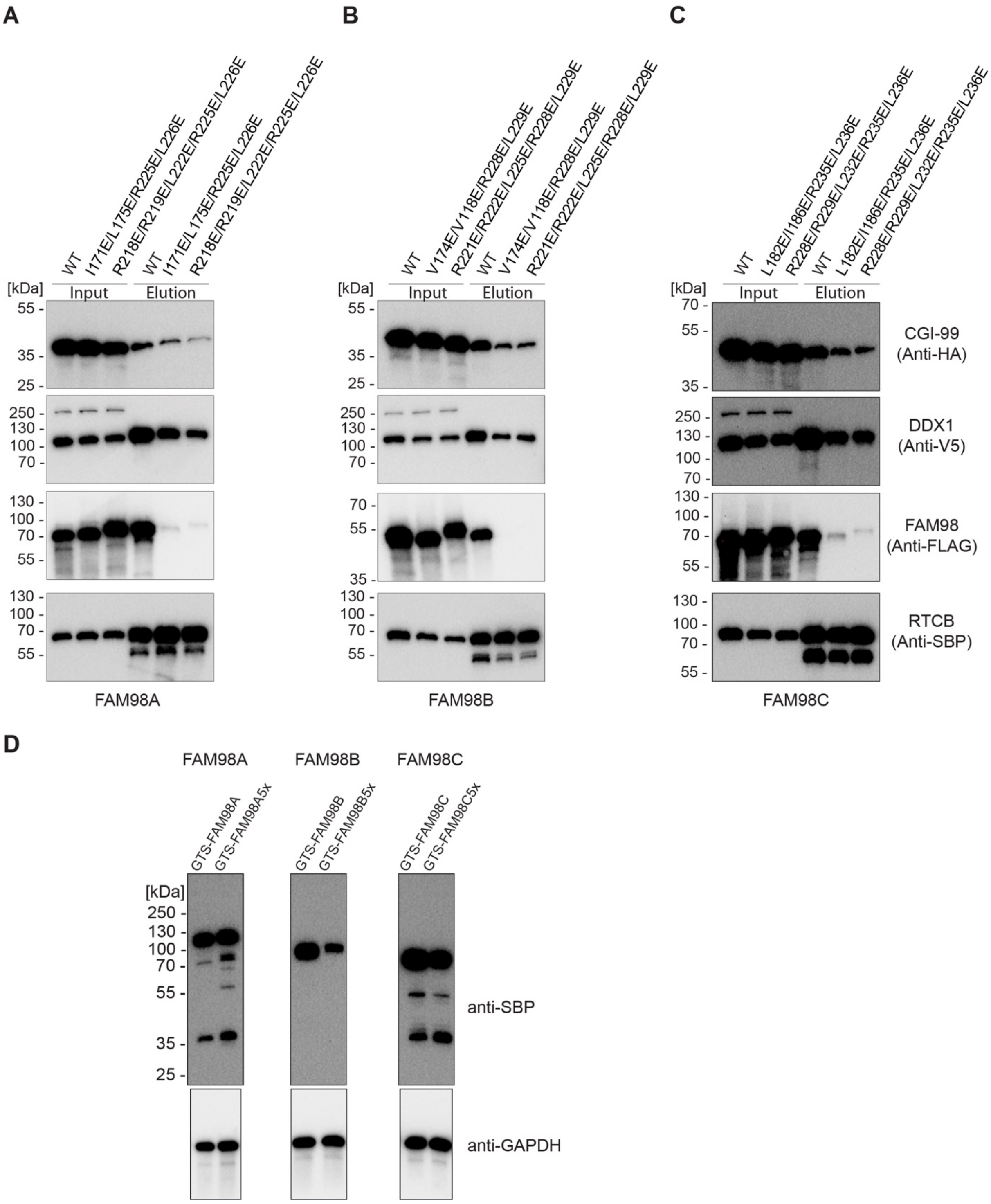

Figure S8 | Validation of FAM98A-C mutant cell lines

(**A-C**) Streptavidin affinity co-precipitation experiments to validate structure-based mutations of FAM98A (**A**) FAM98B (**B**) and FAM98C (**C**) paralogs disrupting recruitment to the tRNA-LC. (**D**) Expression of mEGFP-TEV-SBP- (GTS-) tagged FAM98A-C paralogs in stably transfected FAM98A-C gene knock-out HEK293T cell lines, monitored by Western blot analysis using an anti-SBP antibody. 5x refers to mutants R218E/R219E/L222E/R225E/L226E (FAM98A), R221E/R222E/L225E/R228E/L229E (FAM98B) and R228E/R229E/L232E/R235E/L236E (FAM98C). GAPDH loading controls were run on a separate blot.

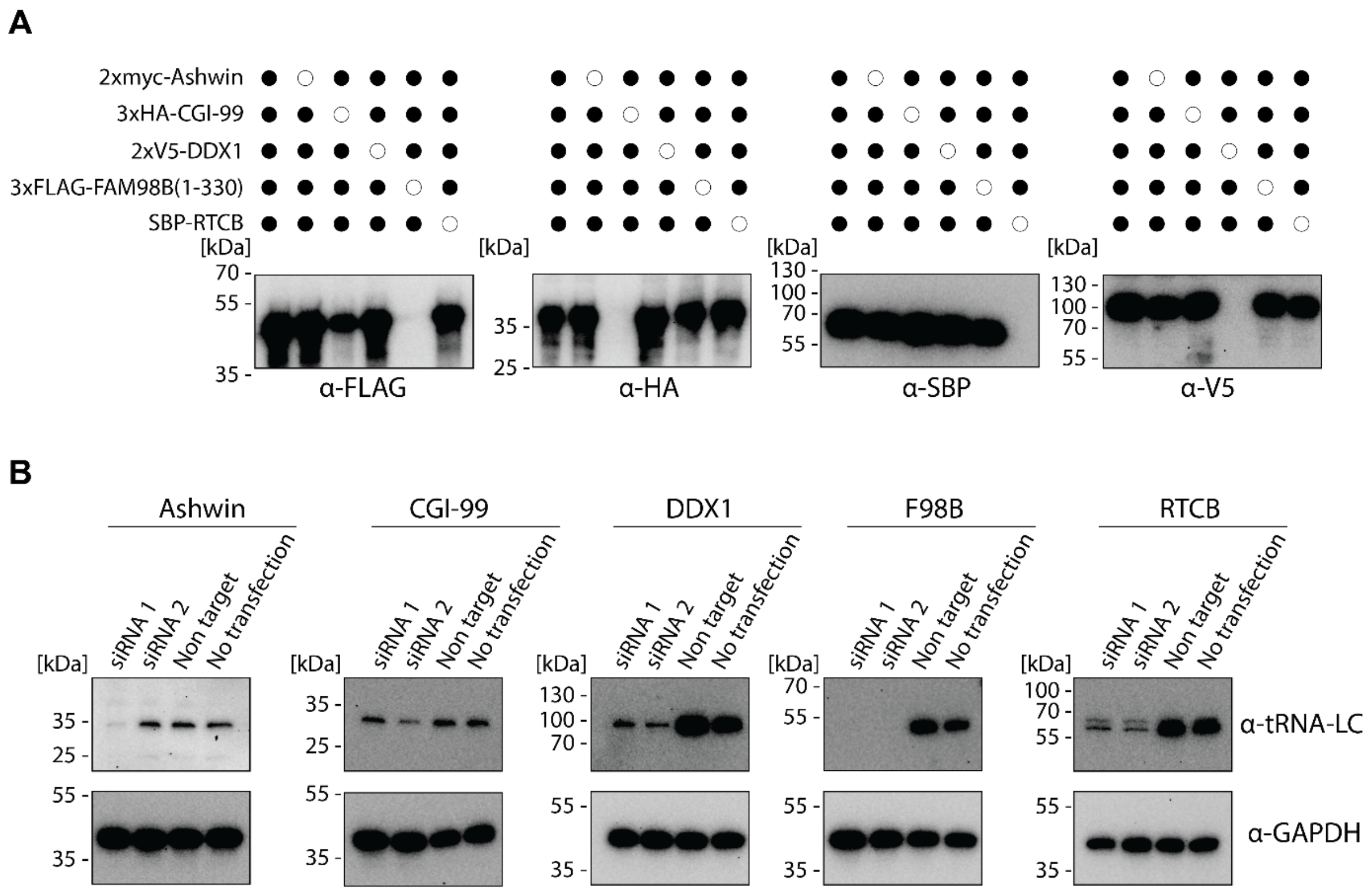

Figure S9 | Antibody validation

(**A**) Co-transfection of tagged tRNA-LC subunits with subsequent immunoblotting. Specific tag subunits were omitted sequentially to confirm antibody specificity. (**B**) siRNA-mediated depletion of tRNA-LC subunits to confirm antibody specificity. GAPDH loading controls were run on a separate blot.
