# Supplementary material for: Structural framework for the assembly of the human tRNA ligase complex
Source: Nat Commun. 2026 Sep 17;17:9883. doi: 10.1038/s41467-026-77450-y (PMC13586351; doi:10.1038/s41467-026-77450-y)
Supplement: Supplementary file 3 [file 41467_2026_77450_MOESM3_ESM.pdf]

nature portfolio

Peer Review File

Structural framework for the assembly of the human tRNA ligase complex

Corresponding Author: Professor Martin Jinek

This file contains all reviewer reports in order by version, followed by all author rebuttals in order by version.

Version 0:

Reviewer comments:

Reviewer #1

(Remarks to the Author)
Pfleiderer et al. present structural and functional characterization of human tRNA ligase complex (tRNA-LC). The subject of this manuscript is timely and interesting. tRNA-LC participates in important processes in the cell, in particular in tRNA maturation, but the understanding of its mechanism of action is far from complete.

The manuscript describes a cryo-EM structure of a truncated five-subunit tRNA-LC as well as a structure of tRNA-LC in complex with PYROXD1 protein which interacts with the catalytic subunit - RTCB. The structures showed that the central element of the complex is a four-helical bundle made by CGI-99, FAM98B (the two proteins form a tight intertwined dimer) and C-terminus of DDX1. This bundle is flanked by globular RTCB and additional domains of CGI-99, FAM98A that form "pincer-like" module. This regions also binds a small globular part of the Ashwin (ASW) subunit.

A valuable element of the manuscript are co-precipitation assays which were used to validate the importance of the interface residues of CGI-99 and FAM98B. Other important results are presented in Fig. 3 and 4. Based on the structure, a mutant of DDX1 was designed which did not interact with the rest of the tRNA-LC. A cell line with the same homozygous mutation had a defect in tRNA maturation. Similar results are presented for tRNA-LC variants in which ASW binding was perturbed. This showed that interactions of DDX1 and ASW with tRNA-LC have functional relevance. The last part of the manuscript shows that FAM98B paralogs – FAM98A and FAM98C can form complexes with tRNA-LC but ASW is absent from these complexes. Cell-based experiments showed that complexes with FAM98A or FAM98C do not participate in tRNA maturation.

The introduction of the paper states that "structural organization of the tRNA-LC remains poorly understood". I fact, a homologous structure of fish tRNA-LC complex was published recently (PMID: 40220997). This structure is largely overlooked in the manuscript, only being mentioned briefly in the discussion section. The authors should compare human and fish structures and comment on the findings in the previously published paper. Such a comparison should, at the very least, be described in the Discussion section.

Inspection of the materials attached to this submission reveals issues with the coordinate files. For the tRNA-LC – PYROXD1 structure, the model provided is not fitted to the EM map, so they could not be deposited together. The attached validation report is not for manuscript review, so it does not come from the final deposition. Inspection of the coordinates of the tRNA-LC – PYROXD1 structure reveals severe steric clashes between RTCB and PYROXD1 subunits. In particular between PYROXD1 residues 441-494 (approximate range) and RTCB residues 433-468. When the structure is fitted into the provided map it is clear that the 433-474 fragment of RTCB is not supported by the density.

The refined model should only comprise parts of the structure that are visible in the density — in other words, parts that are supported by the experimental data. Regions of both structures that are not visible in the potential map should not be included in the atomic model. For tRNA-LC – PYROXD1 structure, for example, this includes residues 269-297 (approximate range) of chain F, residues 134-141 of chain B and other regions. In the tRNA-LC structure the density is missing for residues 435-472 of chain D for example. In both structures, this remark also applies to multiple side chains.

The coordinates of the tRNA-LC – PYROXD1 structure comprise two copies of 1,4-dihydronicotinamide adenine dinucleotide (NAI) ligand. One is located at the active site of RCB and it clashes with PYROXD1. Its use for structure

determination is not described in the manuscript and it is not supported by the density. Perhaps it is a carryover from the models used for initial building of the structure. Although Supplementary Table 1 provides refinement statistics, the paper does not detail the refinement procedure in the Methods section.

Overall, the structural data provided has technical issues.

Minor points:

The preprint: https://www.biorxiv.org/content/10.1101/2023.04.06.535761v1.full.pdf and the published version of this paper (PMID: 40069351) are both cited in this manuscript (references 20, and 32, respectively). Only the peer-reviewed published version should be cited if it is available.

In Fig 2G two antibodies were used for the detection of CGI-99 (anti-CGI-99 and anti-HA) and in Fig. 2H two antibodies were used for the detection of FAM98B (anti-FAM98B and anti-FLAG). This most likely served to distinguish overexpressed proteins from endogenous ones but this is not explained in the text. It also needs to be explained why anti-CGI-99 antibody detects two bands in input fraction. It is also notable that in Fig. 3H different amounts of DDX1 are present in the input and PD fractions for different FAM98B mutants.

Page 6. The end of last paragraph, typo in CGI-99

Fig. 3G, ‘P’ is missing in mutant label.

Adding a schematic of the tRNA species/fragments would make the outcome of the tRNA maturation experiments easier to follow.

The legends for Figures S2 and S3 have been mixed up. Figure S2 appears to present cryo-EM pipeline for RNA-LC complexed with PYROXD1 (not tRNA-LC) and Figure S3 appears to present cryo-EM pipeline for RNA-LC (not PYROXD1 complex).

Reviewer #2

(Remarks to the Author)
The authors present a high-resolution structure of the human tRNA ligation complex, in complex with PYROXD1 (used to stabilize/improve the resolution of RTCB). This is the first atomic resolution structure of the “full” human complex, which follows up on the author’s previous work (lower resolution). It also compliments a recent structure of other metazoan partial tRNA ligation complexes published in JBC in April of 2025. I congratulate the authors on a long-time structural challenge. Through this structure, the authors use the structure to describe the complex’s architecture, including a key helical bundle at the interface with RTCB. The authors also provide novel insights into FAM98B and Ashwin’s role in the tRNA ligation complex, with foreshadowing of a mystery for the roles of FAM98A/C. To support their structures, the authors provide extensive variants of different core components and perform pull-downs of the complex to observe disruption of complex formation. This is important work that advances the field’s understand of the complex assembly and architecture, especially of a human specific complex.

While I can appreciate the value this paper brings to the field, I do have a few concerns, which can be primarily addressed through *revision to the text*.
1. First, to resolve the structure, the authors combine two structures. The way this was done is hinted at, but it not fully conveyed through the text and was only fully understood by looking at the maps/models.
2. Since the authors made a composite model by combining structures, they should deposit maps that all align for easier interpretation by readers. The models should also match the maps. For the tRNA-LC + PYROXD1 map/model, the map is flipped on the Y axis relative to the other models such that they are not aligned when opened. Please correct this.
3. Along these lines, for the tRNA-LC + PRYOXD1 model, as noted by the PDB validation, there is a high level of poorly fit residues within the map. What steps have the authors taken to correct this? I believe additional model fitting can help with this, but regions that do not fit within the density resolved may also need to be removed. I did not see these regions of low resolution discussed. Two areas this reviewer noted are:
a. Chain D 440-463 (helix and sheet are not well fit within density), and Chain Example, at 0.1 contour
b. Another example (contour 0.579), around Chain A, loop around 40:
4. The other maps fit well in this region, so I do not think they should be superimposed for the RTCB region.
5. On the other map, the C-terminus of Chain A and D are poorly fit.
6. In the introduction, the authors said there are 24 intron-containing tRNAs. This should be rephrased as “high confidence” tRNAs. There are 28 tRNAs that contain introns. The ILE genes appear to be overlooked.
7. The introduction is assuming the reader understands this is all human, but they don’t clearly state this.
8. The authors say splicing is important for L-shaped tRNA formation, which is misleading. Previous TSEN + tRNA structures reveal the L-shape pre-splicing.
9. The authors forgot to include the citation when they reference the DDX1 truncation being sufficient for tRNA-LC function.
10. Figure 2: There appear to be some inconsistencies with the CGI-99 blot and the anti-HA. Do the authors have antibody validations?

11. Figure 3, The helices should be labelled. The same amount of the anti DDX1 blot should be shown, as is shown for the V5. Do the authors have any insight why they see the delta-SPRY domain DDX1 in the IP, but not the input for anti DDX1? I am concerned about antibody specificity.
12. In the discussion, the authors mention DDX1 is dispensable for XBP1 ligation – reference should be provided.
13. To the two previous points, validation of all antibodies and cell lines should be shown.
14. Finally, the authors present a fairly detailed Methods section in some regions but have lacking methods in others. Thus, there are some areas for improvement to enhance general rigor and reproducibility. Throughout, there are some inconsistencies in formatting of units (space, no space between – most should have a space) and grammatical errors (including different ways to refer to "milk TBST"). These should be corrected. The authors should revisit this section and ensure robust inclusion of conditions and methods are used throughout. This includes, but is not limited to:
• Inclusion of all antibody information for the Western blots, including validation
• Information on the sequence of Northern blot probes and the methodology used
• Information on CRISPR cell lines, including validation
• More information on particle picking through CryoSparc
• Additional information on model refinement should be provided
• Clear reference to which side of the cryo-EM grids had protein put on them "one side of the grid"
• FC8 should be spelled out
• Additional information on companies used to source reagents, such as the strepavidin resin.

Reviewer #3

(Remarks to the Author)
This paper present new cryo-EM structures of the human tRNA ligase complex that provide insights into complex assembly. Structure-inspired mutagenesis of subunit interfaces identifies key determinants of assembly.

Comments:

Abstract: The pathway of tRNA splicing should not be viewed as "non-canonical". Please delete this term.

Abstract: It is a bit of a misdirection to state that "the molecular mechanisms underpinning tRNA ligation remain elusive." The ligation mechanism of RtcB is very well understood and the present structures do not bear on that topic. Rather, it is the structural architecture of the mammalian ligase complex that is illuminated by the present study. I recommend the sentence "Athough recent studies . . . " should be deleted from the Abstract.

Abstract: In the same vein, suggest deleting "and mechanism" from the final sentence of Abstract. It is more on target to say that the results provide new insights into the assembly of the tRNA ligase complex.

Introduction: Update reference 20 (now published in 2025)

p. 7: Regarding the cell line expressing DDX1-K720P, please delete "marked" from the sentence alluding to accumulation of a 5'-fragment of tRNA-Tyr. Also, the claim that DDX1 is required for proper tRNA maturation, based solely on the analysis on one tRNA species, is belied by the fact that the level of mature tRNA-Tyr is unaffected. (See my comments on the accompanying manuscript).

p. 8: The same issues of interpretation apply to the ASW-P59E cell line (Fig. 8E). The text on p. 8 seems to have a typo (W589P) referring to the P59E mutation.

The Results section on FAM98 paralogs showing that FAM98B recruits ASW is more thematically in tune with the content of the accompanying manuscript and could well be included in that paper. Indeed, the data in Fig. 5E on the FAM98 knockouts reprises the same experiment performed in the accompanying paper, with the addition of a complementation test.

Version 1:

Reviewer comments:

Reviewer #1

(Remarks to the Author)
The authors have responded appropriately to my remarks, and I have no further comments.

Reviewer #2

(Remarks to the Author)
The authors have made or addressed several of the requested changes.
Please note, the Figshare file: 10.6084/m9.figshare.30521411 was not accessible. Thank you for including the information in the supplement.

However, I still have concerns with the models.

The 9SPD model has several regions that do not have sufficient/any density at high resolution for model fitting: 138-153, 445-467. The authors state in the rebuttal that the later is not modeled into the structure since it's in a disordered loop "The RTCB segment spanning residues 433-468 corresponds to a structurally disordered loop that is not modeled in the structure of the RTCB-PYROXD1 complex (PDB: 8ORJ). The loop is also disordered in the structure of the tRLC-PYROXD1 complex presented in this manuscript and not modeled in the revised structure file."
The authors justify this to reviewer number 1, but I disagree, this region should be omitted from the structure. It is more likely that someone will download the structure and not read the paper than the reverse. The published structure should recapitulate the data.

In the other model FAM98B also does not appear to be in a region sufficient for modeling. RTRAF is also poorly modeled. There are remaining issues of poor model fit that can and should be addressed: examples B13,89, C74. As the authors are hoping to make these seminal structures for the field of human tRNA ligation, the models should closely align with the densities.

Reviewer #3

(Remarks to the Author)
The authors have responded to concerns of Reviewers 1 and 2 regarding the discrepancies between the EM maps and the structural models, and the need for additional technical details. I will defer to Reviewers 1 and 2 re whether things are now satisfactory on that score.

Most of my principal concerns have been addressed, with exceptions pertaining to the results in Fig. 3H and 4H.

I objected to the conclusion that “association of DDX1 with RTCG is required for proper tRNA maturation” because the level of mature tRNA-Tyr did not change. The authors responded by citing “previous experiments using temporary depletion of tRNA-LC subunits have not resulted in strong phenotypes indicating loss of mature tRNAs, highlighting the general robustness of the spliced tRNA pool to such perturbations.”

That’s all well and good, but they elide the fact that the experiments in the present study do not entail “temporary depletion” but instead employ a cell line carrying a homozygous K720P mutation in the DDX1 gene, which selectively disrupts DDX1 assembly with the tRNA-LC.”

Thus, there is no issue of “general robustness” beyond the most parsimonious conclusion from the data that the interaction of DDX1 might AFFECT tRNA maturation (evinced by presence of 5’ tRNA fragment in K720P cells) without being REQUIRED for tRNA splicing. Alternatively, there is residual interaction of the DDX1 K720P mutant that is not reflected in the co-IP assays.

The same caveats apply to Fig. 4H that reports a cell line with a homozygous ASW-P59E mutation in which the level of mature tRNA does not change, though a 5’-tRNA fragment is evident.

The text should be revised to rectify this over-reach, by tempering the assertion that the interaction is required.

Also, in light of the revisions to the accompanying manuscript regading improving the Northern blots, the authors should add some comment on the blot here in Fig. 5E depicting tRNA maturation, in which the levels of mature tRNA-Tyr are almost undetectable because the hybridization conditions were not optimal.

Version 2:

Reviewer comments:

Reviewer #2

(Remarks to the Author)
The reviewers have sufficiently addressed my critiques.

Reviewer #3

(Remarks to the Author)
Revisions are satisfactory.

Open Access This Peer Review File is licensed under a Creative Commons Attribution 4.0 International License, which permits use, sharing, adaptation, distribution and reproduction in any medium or format, as long as you give appropriate credit to the original author(s) and the source, provide a link to the Creative Commons license, and indicate if changes were made.
In cases where reviewers are anonymous, credit should be given to 'Anonymous Referee' and the source.
The images or other third party material in this Peer Review File are included in the article's Creative Commons license, unless indicated otherwise in a credit line to the material. If material is not included in the article's Creative Commons license and your intended use is not permitted by statutory regulation or exceeds the permitted use, you will need to obtain permission directly from the copyright holder.
To view a copy of this license, visit https://creativecommons.org/licenses/by/4.0/

Nature Communications manuscript NCOMMS-25-84564A

Structural framework for the assembly of the human tRNA ligase complex

Response to Reviewer Comments

Reviewer #1 (Remarks to the Author):

Pfleiderer et al. present structural and functional characterization of human tRNA ligase complex (tRNA-LC). The subject of this manuscript is timely and interesting. tRNA-LC participates in important processes in the cell, in particular in tRNA maturation, but the understanding of its mechanism of action is far from complete. The manuscript describes a cryo-EM structure of a truncated five-subunit tRNA-LC as well as a structure of tRNA-LC in complex with PYROXD1 protein which interacts with the catalytic subunit - RTCB. The structures showed that the central element of the complex is a four-helical bundle made by CGI-99, FAM98B (the two proteins form a tight intertwined dimer) and C-terminus of DDX1. This bundle is flanked by globular RTCB and additional domains of CGI-99, FAM98A that form "pincer-like" module. This regions also binds a small globular part of the Ashwin (ASW) subunit.
A valuable element of the manuscript are co-precipitation assays which were used to validate the importance of the interface residues of CGI-99 and FAM98B. Other important results are presented in Fig. 3 and 4. Based on the structure, a mutant of DDX1 was designed which did not interact with the rest of the tRNA-LC. A cell line with the same homozygous mutation had a defect in tRNA maturation. Similar results are presented for tRNA-LC variants in which ASW binding was perturbed. This showed that interactions of DDX1 and ASW with tRNA-LC have functional relevance. The last part of the manuscript shows that FAM98B paralogs – FAM98A and FAM98C can form complexes with tRNA-LC but ASW is absent from these complexes. Cell-based experiments showed that complexes with FAM98A or FAM98C do not participate in tRNA maturation.

We thank the Reviewer for the thorough summary and the positive feedback on our study.

The introduction of the paper states that "structural organization of the tRNA-LC remains poorly understood". I fact, a homologous structure of fish tRNA-LC complex was published recently (PMID: 40220997). This structure is largely overlooked in the manuscript, only being mentioned briefly in the discussion section. The authors should compare human and fish structures and comment on the findings in the previously published paper. Such a comparison should, at the very least, be described in the Discussion section.

We acknowledge that the structure of the human tRNA-LC is similar to that of the recently published structure of the homologous zebrafish complex and agree with the Reviewer that the structure should be discussed in more detail. To this end we have revised the manuscript as follows:

In the Introduction, we have revised the fourth paragraph (p. 4, lines 90-98) to state:

"High-resolution structures of human RTCB in complexes with Archease[19] and PYROXD1[20] have been elucidated, revealing the molecular details of their activation and protection mechanisms. However, the structural organization of the human tRNA-LC remains to be elucidated, despite its fundamental importance in RNA biology and disease. Previous structural and biochemical studies have provided insights into the overall composition and interactions within human tRNA-LC[13,19,20], and its architecture has been informed by the recently determined structure of the homologous complex from zebrafish. Yet, critical structural aspects of the human tRNA-LC remain unvalidated, in particular the molecular determinants underpinning complex assembly and the functional roles of auxiliary subunits in modulating complex stability and activity."

In the Results section, the following statements have now been included (p. 6, lines 187-189):

*"Superposition of the human tRNA-LC with the structure of the recently determined zebrafish tRNA-LC reveals a high degree of overall similarity with an RMSD of 0.78 Å over equivalent residues (**Figure S5**)."*

In the Discussion section, we have made the following statement (p. 10, lines 363-365):

"In our study, we present cryo-EM reconstructions of the human tRNA-LC, complementing a recently determined structure of zebrafish tRNA-LC[32], and extend our findings by additional interaction and functional in cellulo assays."

Inspection of the materials attached to this submission reveals issues with the coordinate files. For the tRNA-LC – PYROXD1 structure, the model provided is not fitted to the EM map, so they could not be deposited together. The attached validation report is not for manuscript review, so it does not come from the final deposition.

We are very sorry to realize that we accidentally uploaded the wrong PDB files and validation reports during the manuscript submission process. The PDB files that were mistakenly uploaded reflect the starting models prior to any building and do not correspond to the PDB validation reports.

Based on the Reviewer's feedback, we checked our structures and made additional changes, as outlined in the responses below. The new coordinate files and the corresponding correct validation reports are now included in the submitted revision. We sincerely apologize for our mistake and thank the Reviewers for their understanding.

Inspection of the coordinates of the tRNA-LC – PYROXD1 structure reveals severe steric clashes between RTCB and PYROXD1 subunits. In particular between PYROXD1 residues 441-494 (approximate range) and RTCB residues 433-468.

We apologize to the Reviewer for the confusion caused by accidentally uploading the wrong PDB file that contained a steric clash between RTCB and PYROXD1.

The RTCB segment spanning residues 433-468 corresponds to a structurally disordered loop that is not modeled in the structure of the RTCB-PYROXD1 complex (PDB: 8ORJ). The loop is also disordered in the structure of the tRLC-PYROXD1 complex presented in this manuscript and not modelled in the revised structure file.

When the structure is fitted into the provided map it is clear that the 433-474 fragment of RTCB is not supported by the density.

The cryo-EM map of the tRNA-LC complex (without PYROXD1) suffers from orientation bias due to the preferential location of the tRNA-LC particles at the air-water interface during grid freezing. As a result, part of the RTCB subunit (residues 444-474), which comprises the enzyme's active site, appears to be disordered and missing from the density map. It is a generally established principle in structural biology that the atomic model is the best possible *interpretation* of the experimental data, *in light of prior information*. When the data is known to suffer from an experimental artifact, it is prudent, in our view, not to introduce that artifactual information into the model. In our case, there are multiple high-resolution structural models of RTCB obtained by X-ray crystallography (PDB 7P3B from Kroupova et al., eLife, 2021; PDB 8BTT and others from Kopp et al., Nat Communications, 2024). The structural model of the tRNA-LC was completed using the apo-RTCB structure (PDB 8BTT) as well as the information coming from the experimental data for the tRNA-LC–PYROXD1 complex (for ASW, FAM98B, CGI-99 and DDX1). Omitting the above RTCB residues from the model would imply that a key part of RTCB becomes unfolded upon formation of the tRNA-LC complex, which we believe is not the case. The complex is catalytically active in vitro, as shown in this manuscript and in prior literature (Kroupova et al., eLife, 2021), confirming that the active site of RTCB is intact and not affected by the interactions with the other subunits. For this reason, we would prefer keeping the 433-474 fragment in the tRNA-LC model, as based on the higher-resolution X-ray structures. To address this issue, we have included the following statement in the Methods section (p. 28, lines 636-646):

"The atomic models of the tRNA-LC and PYROXD1-tRNA-LC complexes were built using AlphaFold3 predictions for the ASW, CGI-99, FAM98B and DDX1 subunits together with previously determined crystal structure of RTCB (PDB: 7P3B) and cryo-EM structure of the RTCB-PYRODX1 complex (PDB: 8ORJ) as starting models[30]. The subunits were rigid-body docked into the cryo-EM maps using ChimeraX[41], adjusted manually using Coot (0.9.9.92 EL)[42] and subsequently refined in real space using Phenix[43]. For the tRNA-LC model, the experimental density maps suffer from orientation bias resulting from preferential location of the particles at the air-water interface. For this reason, the tRNA-LC model was completed using structural information from the tRNA-LC–PYROXD1 complex (for ASW, FAM98B,

CGI-99 and DDX1 subunits) and the crystallographic structure of RTCB. Notably, RTCB residues 444-473, which are adjacent to the ligase catalytic site and which are not visible in the biased experimental map are modeled based on the apo-RTCB crystal structure (PDB 8BTT[19]).”

For tRNA-LC – PYROXD1 structure, for example, this includes residues 269-297 (approximate range) of chain F, residues 134-141 of chain B and other regions. In the tRNA-LC structure the density is missing for residues 435-472 of chain D for example. In both structures, this remark also applies to multiple side chains.

We have revised the atomic models of tRNA-LC and tRNA-LC–PYROXD1 to trim the N- and C-termini of the non-catalytic subunits (chains A, B, C and D) and unstructured loops based on the cryo-EM map. Please note that both B-factor sharpened and unsharpened maps were used in the building process to complete the model as best as possible. In the case of the tRNA-LC sample, for which the map suffers from orientation bias, we used structural information from the tRNA-LC-PYROXD1 complex to complete the model.

The coordinates of the tRNA-LC – PYROXD1 structure comprise two copies of 1,4-dihydronicotinamide adenine dinucleotide (NAI) ligand. One is located at the active site of RCB and it clashes with PYROXD1. Its use for structure determination is not described in the manuscript and it is not supported by the density. Perhaps it is a carryover from the models used for initial building of the structure. Although Supplementary Table 1 provides refinement statistics, the paper does not detail the refinement procedure in the Methods section.

We apologize for the error – as stated above, the wrong PDB file was accidentally uploaded with the submitted manuscript. The correct atomic model of the tRNA-LC–PYROXD1 complex, as submitted in the revised version of the manuscript, contains only one NADH molecule.

Overall, the structural data provided has technical issues.

Again, we are sincerely sorry for our mistake and believe that the newly uploaded, correct PDB files have addressed the technical issues raised by the Reviewer.

Minor points:

The preprint: https://www.biorxiv.org/content/10.1101/2023.04.06.535761v1.full.pdf and the published version of this paper (PMID: 40069351) are both cited in this manuscript (references 20, and 32, respectively). Only the peer-reviewed published version should be cited if it is available.

We thank the Reviewer for noticing the error. The revised manuscript only cites the published paper.

In Fig 2G two antibodies were used for the detection of CGI-99 (anti-CGI-99 and anti-HA) and in Fig. 2H two antibodies were used for the detection of FAM98B (anti-FAM98B and anti-FLAG). This most likely served to distinguish overexpressed proteins from endogenous ones but this is not explained in the text. It also needs to be explained why anti-CGI-99 antibody detects two bands in input fraction. It is also notable that in Fig. 3H different amounts of DDX1 are present in the input and PD fractions for different FAM98B mutants.

We thank the Reviewer for pointing out this ambiguity. The two signals for the ani-CGI-99 and anti-FAM98B antibodies correspond to the endogenous and overexpressed (HA- or FLAG-tagged, respectively) proteins. We have addressed this in the figure legend of Figure 2.

“Fig 2 | FAM98B and CGI-99 form an intricate heterodimeric subcomplex. (A) Cartoon representation of the FAM98B/CGI-99 dimer. Colors correspond to the coloring scheme in Fig. 1. The head and interface modules are indicated. (B–F) Close-up views showing key interface residues involved in the CGI-99–FAM98B interaction. (G) Streptavidin affinity co-precipitation of wild-type (WT) and mutant CGI-99 proteins co-expressed with the other tRNA-LC subunits in HEK293T cells. SBP-tagged RTCB was used as bait. The two bands detected by the anti-CGI-99 antibody correspond to the native and the recombinant (HA-tagged) proteins. (H) Streptavidin affinity co-precipitation of wild-type (WT) and mutant FAM98B proteins co-expressed with the other tRNA-LC subunits in HEK293T cells. SBP-tagged RTCB

was used as bait. The two bands detected by the anti-FMA98B antibody correspond to the native and the recombinant (HA-tagged) proteins."

Page 6. The end of last paragraph, typo in CGI-99

The typo has been corrected.

Fig. 3G, 'P' is missing in mutant label.

The label has been corrected.

Adding a schematic of the tRNA species/fragments would make the outcome of the tRNA maturation experiments easier to follow.

The requested schematic has been added to Figure 3G.

The legends for Figures S2 and S3 have been mixed up. Figure S2 appears to present cryo-EM pipeline for RNA-LC complexed with PYROXD1 (not tRNA-LC) and Figure S3 appears to present cryo-EM pipeline for RNA-LC (not PYROXD1 complex).

We thank the Reviewer for spotting the error. The legends have been corrected accordingly.

Reviewer #2 (Remarks to the Author):

The authors present a high-resolution structure of the human tRNA ligation complex, in complex with PYROXD1 (used to stabilize/improve the resolution of RTCB). This is the first atomic resolution structure of the "full" human complex, which follows up on the author's previous work (lower resolution). It also compliments a recent structure of other metazoan partial tRNA ligation complexes published in JBC in April of 2025. I congratulate the authors on a long-time structural challenge. Through this structure, the authors use the structure to describe the complex's architecture, including a key helical bundle at the interface with RTCB. The authors also provide novel insights into FAM98B and Ashwin's role in the tRNA ligation complex, with foreshadowing of a mystery for the roles of FAM98A/C. To support their structures, the authors provide extensive variants of different core components and perform pull-downs of the complex to observe disruption of complex formation. This is important work that advances the field's understand of the complex assembly and architecture, especially of a human specific complex. First, to resolve the structure, the authors combine two structures. The way this was done is hinted at, but it not fully conveyed through the text and was only fully understood by looking at the maps/models.

We thank the Reviewer for appreciating the significance of our work.

We sincerely apologize for the confusion concerning the structural data. We accidentally uploaded the wrong coordinate files during the manuscript submission process. These files correspond to the initial starting models before any manual rebuilding, and not the final refined structures. We are very sorry for the mistake and the inconvenience caused and thank the Reviewer for their understanding.

To generate an atomic model for the tRNA-LC, we used structural information from the available high-resolution crystal structures of RTCB and the cryo-EM structure of the RTCB-PYROXD1 complex. The tRNA-LC model was completed using structural information from the tRNA-LC-PYROXD1 complex for the non-catalytic

Please note that the structures are not actually combined insofar that they represent models built into independently determined cryo-EM maps, with some cross-sample building to improve the quality of the model in part not adequately depicted in the maps. To clarify the issue, we have revised the text in the relevant part of the Results section (p. 5, lines 154-161):

"*While the CGI-99, FAM98B, DDX1 and Ashwin subunits were well resolved, the map lacked definition in a region corresponding to the RTCB active site, due to residual particle orientation bias, precluding the building of a complete atomic model. To address this, we additionally determined a cryo-EM reconstruction of the minimal tRNA-LC complex described above bound to PYROXD1 in the presence of NADH, at a nominal resolution of 3.3 Å (**Fig. 1D,E; Fig. S3,4**). The resulting map had improved local resolution in the RTCB region, enabling us to complete the atomic models of the tRNA-LC and its*

complex with PYROXD1 by iterative building, starting from available crystal structures of RTCB and AlphaFold3 (AF3) structural models of the non-catalytic subunits[29]."

As well as in the Methods section (p 28):

"For the tRNA-LC model, the experimental density maps suffer from orientation bias resulting from preferential location of the particles at the air-water interface. For this reason, the tRNA-LC model was completed using structural information from the tRNA-LC–PYROXD1 complex (for ASW, FAM98B, CGI-99 and DDX1 subunits) and the crystallographic structure of RTCB. Notably, RTCB residues 444-473, which are adjacent to the ligase catalytic site and which are not visible in the biased experimental map are modeled based on the apo-RTCB crystal structure (PDB 8BTT [19])."

Since the authors made a composite model by combining structures, they should deposit maps that all align for easier interpretation by readers. The models should also match the maps. For the tRNA-LC + PYROXD1 map/model, the map is flipped on the Y axis relative to the other models such that they are not aligned when opened. Please correct this.

We apologize for the mistake that led to the wrong coordinate files being uploaded with the maps during the initial submission. We have provided the correct coordinate files together with the associated maps in the revised manuscript. The map files have also been flipped now so that they align with the atomic models. We agree with the Reviewer that it would be easier for the reader to have both maps and models superimposable. However, this is not straightforward. During cryo-EM processing resolution estimation is measured by cross-correlating half maps and using density exclusion volumes (masks) to restrict the impact of noise from the 3D-box. While it is certainly possible to overlay the main maps it would be necessary to translocate the associated half-maps and mask in exactly the same way to achieve identical map quality parameters during refinement. At this point we are not aware of a reliable method to achieve this. Although map suprposition is possible in Chimera, this necessitates resampling of the volumes and results in loss of information. For these reasons, we prefer using (and provide) the originally calculated maps for the tRNA-LC and PYROXD1-tRNA-LC, even though they are not directly superimposable.

Along these lines, for the tRNA-LC + PRYOXD1 model, as noted by the PDB validation, there is a high level of poorly fit residues within the map. What steps have the authors taken to correct this? I believe additional model fitting can help with this, but regions that do not fit within the density resolved may also need to be removed. I did not see these regions of low resolution discussed. Two areas this reviewer noted are:

a. Chain D 440-463 (helix and sheet are not well fit within density), and Chain Example, at 0.1 contour

This region was included in the coordinate file that was mistakenly uploaded with the initial submission but is actually omitted from the final atomic model of the tRNA-LC-PYROXD1 complex. Of note, the region is structurally disordered in the cryo-EM structure of the RTCB-PYROXD1 complex (PDB 8ORJ). We apologize for the confusion and thank the Reviewer for their understanding.

The same region, which covers part of the RTCB catalytic site is also not well-resolved in the density map for the tRNA-LC. However, we would argue in favor of retaining the segment in the structural model of the tRNA-LC. As explained in our response to a similar comment from Reviewer #1 above, the dataset suffers from severe preferred orientation, most likely arising from air–water interface interactions, which are known to compromise protein integrity in cryo-EM. As a result, we modelled this region based on high-resolution crystal structures of RTCB, where this region is ordered. Removing the segment would otherwise misleadingly imply that the RTCB active site is intrinsically disordered in the tRLC complex, which is not the case, given that the complex is catalytically active. For this reason, we prefer to keep the region modeled, while clearly stating that it is not supported by the EM density. To reflect this, we have revised the Methods section of the manuscript as follows:

"The atomic models of the tRNA-LC and PYROXD1-tRNA-LC complexes were built using AlphaFold3 predictions for the ASW, CGI-99, FAM98B and DDX1 subunits together with previously determined crystal structure of RTCB (PDB: 7P3B) and cryo-EM structure of the RTCB-PYRODX1 complex (PDB: 8ORJ) as starting models[29]. The subunits were rigid-body docked into the cryo-EM maps using

ChimeraX[41], adjusted manually using Coot (0.9.9.92 EL)[42] and subsequently refined in real space using Phenix[43]. For the tRNA-LC model, the experimental density maps suffer from orientation bias resulting from preferential location of the particles at the air-water interface. For this reason, the tRNA-LC model was completed using structural information from the tRNA-LC–PYROXD1 complex (for ASW, FAM98B, CGI-99 and DDX1 subunits) and the crystallographic structure of RTCB. Notably, RTCB residues 444-473, which are adjacent to the ligase catalytic site and which are not visible in the biased experimental map are modeled based on the apo-RTCB crystal structure (PDB 8BTT)."

b. Another example (contour 0.579), around Chain A, loop around 40:

The residues 37-41 have been removed from chain A.

The other maps fit well in this region, so I do not think they should be superimposed for the RTCB region.

On the other map, the C-terminus of Chain A and D are poorly fit.

We have trimmed the N- and C-termini of chains A and D in the tRNA-LC complex structure.

In the introduction, the authors said there are 24 intron-containing tRNAs. This should be rephrased as "high confidence" tRNAs. There are 28 tRNAs that contain introns. The ILE genes appear to be overlooked.

We thank the Reviewer for pointing this out. We have revised the introduction (p. 3, lines 52-54) as follows:

"The human genome encodes 416 tRNA genes, 28 of which contain introns. This group includes five Arg (TCT), five Leu (CAA), five Ile (TAT), and thirteen Tyr (GTA) tRNA genes[3,4]."

The introduction is assuming the reader understands this is all human, but they don't clearly state this.

The word "human" was included in the title of the submitted manuscript and mentioned several times in the abstract. Nevertheless, to emphasize this point, we have now revised the introduction section to state the following:

"High-resolution structures of human RTCB in complexes with Archease [19] and PYROXD1 [20] have been elucidated, revealing the molecular details of their activation and protection mechanisms. However, the structural organization of the human tRNA-LC remains to be elucidated, despite its fundamental importance in RNA biology and disease. Previous structural and biochemical studies have provided insights into the overall composition and interactions within human tRNA-LC[13,19,20], and its architecture has been informed by the recently determined structure of the homologous complex from zebrafish. Yet, critical structural aspects of the human tRNA-LC remain unvalidated, in particular the molecular determinants underpinning complex assembly and the functional roles of auxiliary subunits in modulating complex stability and activity. Furthermore, the existence of multiple paralogs of the tRNA-LC subunit FAM98B raises intriguing questions about their potential functional specialization and redundancy within human cells."

The authors say splicing is important for L-shaped tRNA formation, which is misleading. Previous TSEN + tRNA structures reveal the L-shape pre-splicing.

We thank the Reviewer for pointing out this error. We have revised the statement (p. 3, lines 54-56) as follows:

" In all cases, the intron is located one nucleotide 3' to the anticodon. Correct intron removal and exon ligation are essential for tRNA maturation as they ensure correct formation of the anticodon loop, which is critical for both for tRNA charging and decoding during translation."

The authors forgot to include the citation when they reference the DDX1 truncation being sufficient for tRNA-LC function.

We thank the Reviewer for noticing the mistake. Kroupova, A. et al. (2021) has been inserted as a reference.

Figure 2: There appear to be some inconsistencies with the CGI-99 blot and the anti-HA. Do the authors have antibody validations?

We appreciate the careful examination of our co-precipitation assays as unspecific epitope recognition by antibodies is indeed a problem and well documented in literature. We have provided additional evidence as to the specificity of our antibodies in this response and as Supplementary Figure 7.

The Anti-CGI-99 antibody used in this study is a polyclonal antibody. However, it is not known which epitope(s) are being recognized. Considering that we work with many different mutant variants of CGI-99, it is conceivable that the main epitope is disrupted in one of the mutants, preventing recognition. To account for this possibility, we base our conclusion solely on the results of the HA-antibody co-precipitation. The Anti-CGI-99 Western blot mainly serves as a means to judge the relative expression levels of the native and recombinant CGI-99 proteins present in the cells.

We have added uncropped images of all Western blots used in this study to Figshare for Reviewer's reference. The images confirm that each antibody recognizes only a single band in the expected region.

Figure 3, The helices should be labelled.

We would prefer not to introduce helix numbering in the current figures. While such nomenclature can be highly informative for extensively characterized proteins or protein domains, none of the proteins in this study has been structurally characterized previously. In this context, assigning helix numbers would not convey additional mechanistic insight and may instead introduce unnecessary complexity, potentially distracting from the key structural features highlighted in the figures.

The same amount of the anti DDX1 blot should be shown, as is shown for the V5.

We have expanded the relevant blot images in Figure 3F to cover the same area as shown for the Anti-V5 antibody.

Do the authors have any insight why they see the delta-SPRY domain DDX1 in the IP, but not the input for anti DDX1? I am concerned about antibody specificity.

We thank the Reviewer for a thorough examination of our co-precipitation experiments. We would like to note that the delta-SPRY DDX1 construct is detectable in the input fraction (shown below, marked with arrow 1 in both the unadjusted and brightened images), albeit at very low levels compared to the endogenous full-length DDX1. This indicates that the truncated protein represents only a small fraction of the total DDX1 content in the cells. Similarly, the delta-helicase variant is also detectable by the antibody (arrow 2). The anti-DDX1 antibody is polyclonal, which may explain some of the differences in

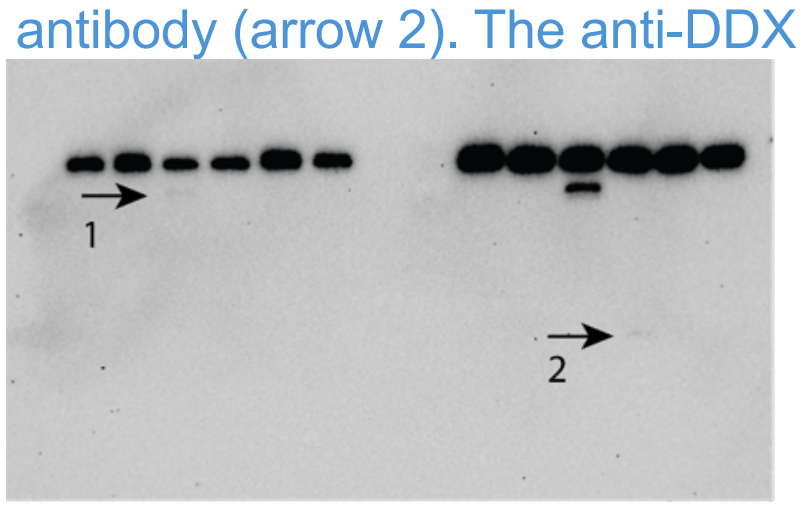

as shown in Figure

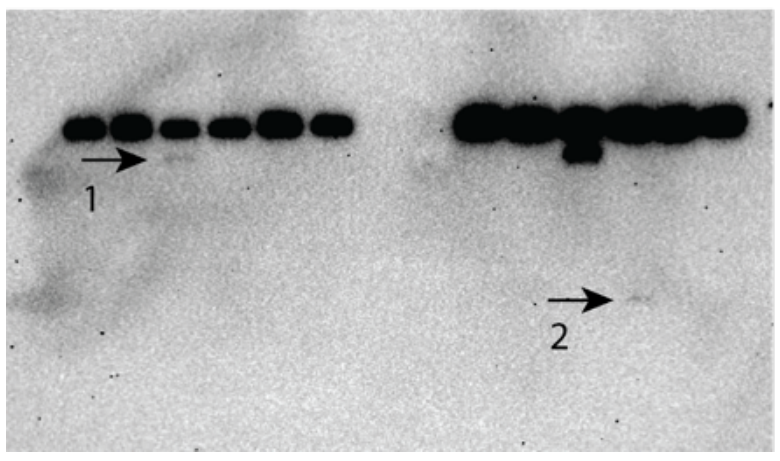

Contrast adjusted

visualizing different DDX1 fragments. Given that we detect the same band with for recombinant protein with the Anti-FLAG antibody and the Anti-DDX1 antibody, we conclude that the Anti-DDX1 antibody is specific. We uploaded all the corresponding WB raw files on Figshare (10.6084/m9.figshare.30521411) for Reviewer's reference.

In the discussion, the authors mention DDX1 is dispensable for XBP1 ligation – reference should be provided.

We thank the Reviewer for noticing the omission. We have added the correct reference (Suzuki, T. *et al.* DDX1 is required for non-spliceosomal splicing of tRNAs but not of XBP1 mRNA. *Commun. Biol.* **8**, 92 (2025)).

To the two previous points, validation of all antibodies and cell lines should be shown.

Concerning the validation of the genome-edited cell lines *DDX1*(K720P) and *ASW*(P59E), Sanger sequencing files have been added to the supplementary data (Figure S5). The raw files have been uploaded to Figshare (10.6084/m9.figshare.30521411).

For the validation of the commercial antibodies for standard epitope tags (FLAG, HA, SBP and V5), we have co-transfected all full-length tRNA-LC subunit expression plasmids as used in the manuscript, systematically omitting one subunit at a time. The resulting Western blot data is included in the revised manuscript as Supplementary Figure 7 and shown below for Reviewer's reference. We observe an unspecific signal for the Anti-V5 antibody at very long exposure at over 250 kDa. This band does not overlap with any of the other subunit bands (e.g. DDX1) and so does not interfere with interpretation of the experiments.

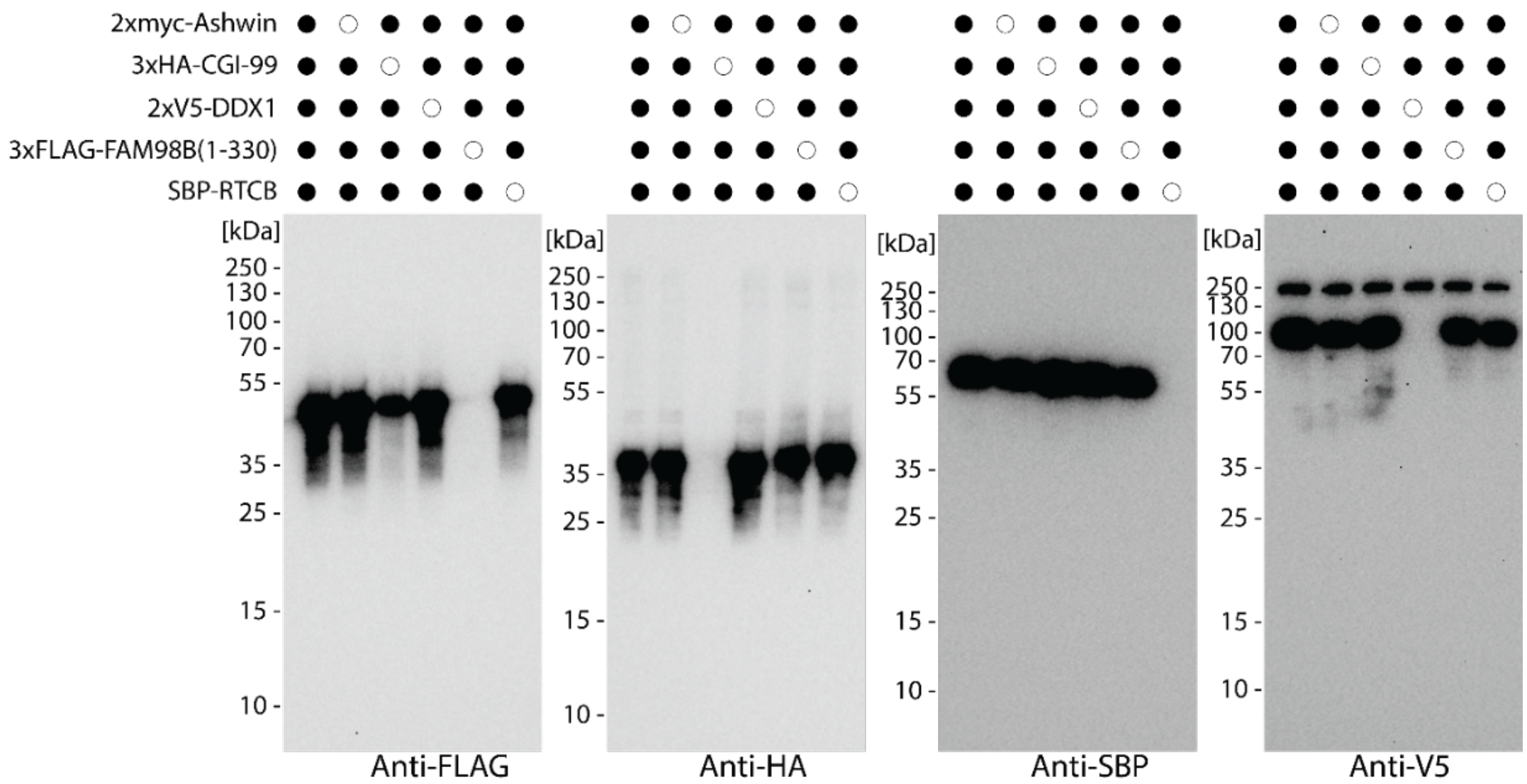

In order to validate the specificity of the protein-specific antibodies we performed siRNA-mediated knockdown of each tRNA-LC subunit in HEK293T cells. The data is now included in the revised manuscript as Supplementary Figure S7 and shown below for Reviewer's reference. We observe complete loss or at least partial reduction in the Western blot signal in the appropriate MW range for at least one of the two siRNAs designed to target the mRNA encoding each subunit.

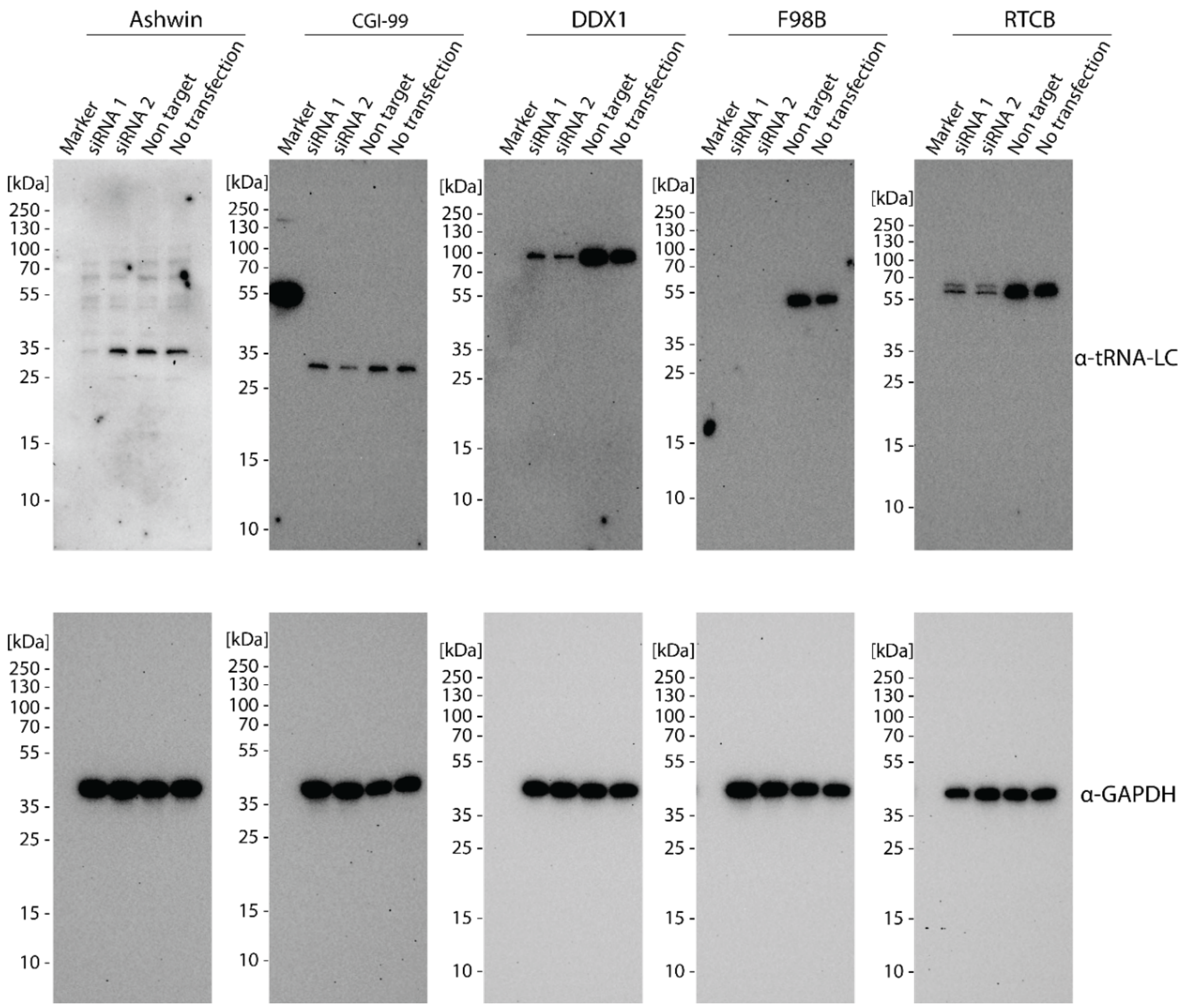

Finally, the authors present a fairly detailed Methods section in some regions but have lacking methods in others. Thus, there are some areas for improvement to enhance general rigor and reproducibility. Throughout, there are some inconsistencies in formatting of units (space, no space between – most should have a space) and grammatical errors (including different ways to refer to "milk TBST"). These should be corrected. The authors should revisit this section and ensure robust inclusion of conditions and methods are used throughout. This includes, but is not limited to:

We thank the Reviewer for the carefully checking our methods section. Units and formatting have been corrected and unified. We use "5% milk-TBST" throughout.

- Inclusion of all antibody information for the Western blots, including validation

We have previously provided antibody information in the reporting summary. As requested, the information been added in the Methods section (p. 24), reproduced below for Reviewer's reference:

*"The following antibodies were used: Anti-HA (antibodyregistry.org, 19.01.2026, AB_390917)[38], Anti-FLAG (antibodyregistry.org, 19.01.2026, AB_439702), Anti-V5 (antibodyregistry.org, 19.01.2026, AB_2537645), Anti-myc (antibodyregistry.org, 19.01.2026, AB_2533008), goat Anti-mouse (antibodyregistry.org, 19.01.2026, AB_2534745), goat Anti-rabbit (antibodyregistry.org, 19.01.2026, AB_258434), anti-CGI-99/RTRAF (antibodyregistry.org, 16.04.2026, AB_2638761), anti-DDX1 (antibodyregistry.org, 16.04.2026, AB_451046), anti-FAM98B (antibodyregistry.org, 16.04.2026, AB_2891713), anti-RTCB (antibodyregistry.org, 16.4.2026, AB_2631474).The anti-Ashwin antibody was generated by the Monoclonal Antibody Facility (MAF) of the Max Perutz Labs in Vienna. The mice were immunized with purified recombinant full-length human ASW, expressed in E. coli. Specificity of all used antibodies was verified experimentally (**Fig. S7**)."*

As for the validation to antibody specificity, please see our response to the previous comments.

- Information on the sequence of Northern blot probes and the methodology used

The experimental details of the Northern blotting experiments have been included in the Methods section (p. 23-24), and provided below for Reviewer's reference.

"Total RNA was isolated from cells using TRIzol (Thermo, 15596018), following the manufacturer's instructions. The RNA samples were mixed with an equal volume of 2x FA buffer (90 % Formamide, 50 mM EDTA pH 8.0, 0.025 % Bromophenol blue w/v, 0.025 % Xylene cyanol w/v), boiled at 95 °C for 3 min and then separated by 10 % UREA-PAGE (National Diagnostics, EC-833), using 0.5x TBE as running buffer (65 mM Tris pH 7.6, 22.5 mM boric acid, 1.25 mM EDTA). The gels were then blotted onto Amersham Hybond-N+ nylon membranes (Cytiva, RPN303B) at a constant current of 250 mA for 3 h, using 0.5x TBE as transfer buffer. After the transfer, the RNA was UV-crosslinked to the membrane and the membrane was blocked in 30 mL pre-heated hybridization buffer [20 mM sodium phosphate pH 7.2, 75 mM sodium citrate, 750 mM NaCl, 7 % SDS w/v, 3 mg sonicated salmon sperm DNA (Thermo, AM9680)] for 1 h, rotating at 50 °C. Radiolabelled DNA probes were added to the hybridization buffer and the membranes were incubated ON rotating at 50 °C. The hybridization solution was discarded and the membranes were washed twice for 1 min in pre-heated (50 °C) low stringency wash buffer (75 mM sodium citrate pH 7.0, 750 mM NaCl, 5 % SDS w/v) and then for 1 min in pre-heated (50 °C) high stringency wash buffer (15 mM sodium citrate pH 7.0, 150 mM NaCl, 1 % SDS w/v). For visualization of the radioactive signal, phosphor screens were exposed to the Northern blots and subsequently imaged using an Amersham Typhoon biomolecular imager. The following oligonucleotides were used as probes: NB_probe_Tyr_5p, 5'-CTACAGTCCTCCGCTCTACC-3'; NB_probe_Gly_5p, TACCACTGAACCACCAATGC; NB_probe_U6 GCAGGGGCCACGCTAATCTTCTCTG).»

- Information on CRISPR cell lines, including validation

The Methods section has been updated with a detailed description of the genome engineering protocols (p. 24-26). The validation has been addressed above in our response to the Reviewer's previous point.

More information on particle picking through CryoSparc

The Methods section has been updated with the requested information (p. 26-28).

Additional information on model refinement should be provided

The Methods section has been updated with the requested information (p. 28), also in response to a similar request from Reviewer #1.

- Clear reference to which side of the cryo-EM grids had protein put on them "one side of the grid"

The information was added to the section "Sample vitrification for cryo-EM analysis"

"UltrAUfoil 300 1.2/1.3 or Quantifoil AU 300 1.2/1.3 grids were glow discharged for 30 s at 15 mA at 28 mbar. Samples were was diluted to 0.3 mg/ ml 1.5 – 2.5 µl were applied to the top side of the grid."

- FC8 should be spelled out

We have added full name of FC8 in the relevant paragraph of the Methods section (p. 26).

- Additional information on companies used to source reagents, such as the strepavidin resin.

The details pertaining to reagent sources have been added in the Methods section.

Reviewer #3 (Remarks to the Author):

This paper present new cryo-EM structures of the human tRNA ligase complex that provide insights into complex assembly. Structure-inspired mutagenesis of subunit interfaces identifies key determinants of assembly.

We thank the Reviewer for the appreciation of our study.

Comments:

Abstract: The pathway of tRNA splicing should not be viewed as “non-canonical”. Please delete this term.

We take the Reviewer’s point. “Non-canonical” has been replaced with “spliceosome independent” in the abstract.

Abstract: It is a bit of a misdirection to state that “the molecular mechanisms underpinning tRNA ligation remain elusive.” The ligation mechanism of RtcB is very well understood and the present structures do not bear on that topic. Rather, it is the structural architecture of the mammalian ligase complex that is illuminated by the present study. I recommend the sentence “Athough recent studies … “ should be deleted from the Abstract.

The relevant sentence in the abstract has been updated as follows:

“The molecular architecture of the human tRNA-LC and the function of the non-catalytic subunits remain elusive.”

Abstract: In the same vein, suggest deleting “and mechanism” from the final sentence of Abstract. It is more on target to say that the results provide new insights into the assembly of the tRNA ligase complex.

The word “mechanism” has been removed from the abstract. The last sentence of the abstract now reads as follows:

“Together, our results provide new insights into the molecular assembly of the tRNA ligase complex, shedding light on its functions in tRNA biogenesis and beyond.”

Introduction: Update reference 20 (now published in 2025)

We thank the Reviewer for noticing the oversight. Reference 20 has been updated.

p. 7: Regarding the cell line expressing DDX1-K720P, please delete “marked” from the sentence alluding to accumulation of a 5’-fragment of tRNA-Tyr. Also, the claim that DDX1 is required for proper tRNA maturation, based solely on the analysis on one tRNA species, is belied by the fact that the level of mature tRNA-Tyr is unaffected. (See my comments on the accompanying manuscript).

We thank the Reviewer for the comment. We have removed “marked” from the relevant sentence. We agree with the Reviewer that the levels of mature tRNA-Tyr do not appear to be affected by the DDX1 mutation. This is most likely due to the general stability of the mature tRNAs, which is the dominant factor determining their steady state levels. Previous experiments using temporary depletion of tRNA-LC subunits have not resulted in strong phenotypes indicating loss of mature tRNAs, highlighting the general robustness of the spliced tRNA pool to such perturbations. However, there is a clear accumulation of the 5’ unspliced tRNA fragment, which we interpret as an indication of a processing defect. Furthermore, the minimal reduction of mature tRNA levels is in line with previously published data (Yang J, et al.. Polyglycine-mediated aggregation of FAM98B disrupts tRNA processing in GGC repeat disorders. Science. 2025 Jul 17;389(6757):eado2403. doi: 10.1126/science.ado2403. PMID: 40674500). We have reformulated the statement as follows:

*“This experiment revealed defective tRNA^{Tyr} biogenesis, as evidenced by the accumulation of unspliced tRNA 5’ fragments, in the K720P mutant line, as compared to wild-type cells (**Fig. 3G**). This indicates that the association of DDX1 with RTCB within tRNA-LC is required for proper tRNA maturation.”*

p. 8: The same issues of interpretation apply to the ASW-P59E cell line (Fig. 8E). The text on p. 8 seems to have a typo (W589P) referring to the P59E mutation.

The typo has been corrected. We refer the Reviewer to our response above concerning the tRNA processing defect in the *ASW* P59E cells.

The Results section on FAM98 paralogs showing that FAM98B recruits ASW is more thematically in tune with the content of the accompanying manuscript and could well be included in that paper. Indeed,

the data in Fig. 5E on the FAM98 knockouts reprises the same experiment performed in the accompanying paper, with the addition of a complementation test.

We acknowledge the overlap of the Pfleiderer et al. and Leitner et al. studies concerning the FAM98 paralogs. We nevertheless believe that these studies focus on distinct aspects of the FAM98 paralogs, with the Leitner study highlighting their functional divergence, while this study focuses on the underpinning structural basis. We believe that the complementation experiments presented in Fig. 5E are critical for validation of the functional data from a structural perspective and strengthen the conclusions of this study. This, however, requires that the FAM98 knock-out data is reprised as controls for the complementation experiments.

Nature Communications manuscript NCOMMS-25-84564B

Structural framework for the assembly of the human tRNA ligase complex

Response to Reviewer Comments

Reviewer #1 (Remarks to the Author):

The authors have responded appropriately to my remarks, and I have no further comments.

We thank the Reviewer for the positive assessment of our manuscript revisions.

Reviewer #2 (Remarks to the Author):

The authors have made or addressed several of the requested changes. Please note, the Figshare file: 10.6084/m9.figshare.30521411 was not accessible. Thank you for including the information in the supplement.

However, I still have concerns with the models.

We thank the Reviewer for the critical feedback. We have made our best effort to address the structural models accordingly. However, we would like to avoid the situation where the models are unnecessarily compromised by experimental artifacts coming from the cryo-EM data, given the availability of high-resolution structural models for RTCB and the RTCB-PYROXD1 complex.

We have updated and tested all links: https://doi.org/10.6084/m9.figshare.32103334

The 9SPD model has several regions that do not have sufficient/any density at high resolution for model fitting: 138-153, 445-467. The authors state in the rebuttal that the later is not modeled into the structure since it's in a disordered loop "The RTCB segment spanning residues 433-468 corresponds to a structurally disordered loop that is not modeled in the structure of the RTCB-PYROXD1 complex (PDB: 8ORJ). The loop is also disordered in the structure of the tRLC-PYROXD1 complex presented in this manuscript and not modeled in the revised structure file."

We have made additional revisions to the structural model of the tRNA-LC (PDB: 9SPD). We have removed a part of the 138-153 segment to improve the fit with the maps.

As for the segment spanning residues 446-468, we would prefer keeping it in the atomic model. The point is that this segment is missing from the map not because it is intrinsically unstructured but because the cryo-EM map suffers from an experimental artifact in this region due to preferential localization of the particles at the solvent-air interface during vitrification and the resulting particle orientation bias. We would like to note that the segment is well structured in the high-resolution (2.6 Å) crystallographic structure of human RTCB in the apo form (PDB: 8BTT) and it is not involved in the formation of crystal contacts in that structure, indicating that it is intrinsically ordered. There is good reason to believe that this segment is also structurally ordered in context of the tRNA-LC. We therefore believe that rigid-body docking of the 8BTT structure to model the missing segment would be justifiable in this case.

We apologize the for the somewhat confusing formulation in our previous response but we would note that the segment is displaced by the interaction of RTCB with PYRODX1 (otherwise there would be a steric clash with PYROXD1) and is therefore disordered *only* in that structure (PDB: 8ORJ). Accordingly, we do not include the segment in the atomic model of tRNA-LC-PYROXD1 complex (PDB: 9SPE).

Nevertheless, as the Reviewer's position seems to indicate that the inclusion of the 433-468 segment might preclude the manuscript from publication, we have decided to delete segment from the model. The new coordinate file (PDB: 31FW) and the corresponding PDB validation report are provided along with the submitted revised version of the manuscript.

The authors justify this to reviewer number 1, but I disagree, this region should be omitted from the structure. It is more likely that someone will download the structure and not read the paper than the reverse. The published structure should recapitulate the data.

We believe that removal of the segment would have the opposite effect. When downloaded without the contextual information provided in the paper, the model would lead to the mistaken interpretation that the catalytic site of RTCB is structurally disordered in the tRNA-LC, implying that the assembly of RTCB with the other non-catalytic subunits somehow leads to unstructuring of the RTCB active site. This contradicts the experimental observation that the RTCB subunit is catalytically active in the context of the tRNA-LC holocomplex.

In the other model FAM98B also does not appear to be in a region sufficient for modeling. RTRAF is also poorly modeled. There are remaining issues of poor model fit that can and should be addressed: examples B13,89, C74.

We used both the B-factor sharpened and unsharpened maps in the process of model building and cycled between the maps so as to obtain as complete models as possible. The certified PDB validation reports provided in the last review round are based on the sharpened maps, which were submitted as the reference maps for the structures. As is commonly the case with cryo-EM data processing, the B-factor sharpening step has improved certain parts of the maps but also made other parts of the density worse, even though these regions are sufficiently ordered in the unsharpened maps to allow model building. For this reason, we include the residues mentioned above in the model for the PYROXD1-tRNA-LC complex. For Reviewer's reference, we have now included the validation report with the unsharpened map used as the reference for the model-to-map quality scores.

As the authors are hoping to make these seminal structures for the field of human tRNA ligation, the models should closely align with the densities.

We agree with the Reviewer that the structural models should reflect the experimental data as best as possible for the sake of a correct scientific record. However, we believe that this should be done without introducing experimental artifacts into the structural models and that this is only possible if the structural models take prior information into account when interpreting the experimentally obtained density maps.

Reviewer #3 (Remarks to the Author):

The authors have responded to concerns of Reviewers 1 and 2 regarding the discrepancies between the EM maps and the structural models, and the need for additional technical details. I will defer to Reviewers 1 and 2 re whether things are now satisfactory on that score. Most of my principal concerns have been addressed, with exceptions pertaining to the results in Fig. 3H and 4H.

We thank the Reviewer for the positive assessment of our manuscript revisions and the critical feedback.

I objected to the conclusion that "association of DDX1 with RTCG is required for proper tRNA maturation" because the level of mature tRNA-Tyr did not change. The authors responded by citing "previous experiments using temporary depletion of tRNA-LC subunits have not resulted in strong phenotypes indicating loss of mature tRNAs, highlighting the general robustness of the spliced tRNA pool to such perturbations." That's all well and good, but they elide the fact that the experiments in the present study do not entail "temporary depletion" but instead employ a cell line carrying a homozygous K720P mutation in the DDX1 gene, which selectively disrupts DDX1 assembly with the tRNA-LC." Thus, there is no issue of "general robustness" beyond the most parsimonious conclusion from the data that the interaction of DDX1 might AFFECT tRNA maturation (evinced by presence of 5' tRNA fragment in K720P cells) without being REQUIRED for tRNA splicing. Alternatively, there is residual interaction of the DDX1 K720P mutant that is not reflected in the co-IP assays. The same caveats apply to Fig. 4H that reports a cell line with a homozygous ASW-P59E mutation in which the level of mature tRNA does not change, though a 5'-tRNA fragment is evident.

The text should be revised to rectify this over-reach, by tempering the assertion that the interaction is required.

We have revised the manuscript text to temper the conclusions concerning the strict requirement of the ASW and DDX1 interactions for pre-tRNA processing.

In the Results section, p. 7, lines 205-211:

"*To test whether the function of DDX1 in pre-tRNA splicing is dependent on its interaction with RTCB within tRNA-LC, we genome-edited HEK293T cells to carry a homozygous K720P-encoding mutation in the DDX1 gene, which selectively disrupts DDX1 assembly within the tRNA-LC and analyzed tRNA^{Tyr} processing using Northern blotting. This experiment revealed the accumulation of unspliced tRNA^{Tyr} 5' fragments in the K720P mutant line, as compared to wild-type cells, indicative of defective tRNA^{Tyr} maturation (**Fig. 3G**). These results imply that maturation of spliced tRNAs is perturbed in the absence of a functional association of DDX1 with RTCB within tRNA-LC.*"

and p. 8, lines 232-237:

"*To test whether the incorporation of ASW in the tRNA-LC affects pre-tRNA splicing, we generated a homozygous mutant HEK293T cell line encoding a P59E substitution in the ASW gene, designed to specifically disrupt its recruitment to the tRNA-LC. Northern blotting analysis revealed accumulation of unspliced tRNA^{Tyr} fragments in the genome-edited cells, indicating that that efficient pre-tRNA splicing is dependent on the assembly of ASW into tRNA-LC. This points to the functional importance of the of the pentameric tRNA-LC holo-complex for tRNA biogenesis..*"

And in the Discussion section

p. 10, lines 320-322:

"*We show that disrupting the recruitment of DDX1 to the tRNA-LC by single point mutations in the C-terminal helix leads to the accumulation of misprocessed tRNA fragments, indicating that the physical association of RTCB and DDX1 contributes to the efficiency of pre-tRNA splicing. Notably, prior studies have shown that while DDX1 is required pre-tRNA processing, it appears to be dispensable for XBP1 mRNA splicing*[31]*. In light of our findings, this implies that the physical integrity of tRNA-LC is important in the context of the nuclear function of RTCB in tRNA biogenesis but is not critical for its cytoplasmic role in the unfolded protein response.*"

Also, in light of the revisions to the accompanying manuscript regading improving the Northern blots, the authors should add some comment on the blot here in Fig. 5E depicting tRNA maturation, in which the levels of mature tRNA-Tyr are almost undetectable because the hybridization conditions were not optimal.

We acknowledge the Reviewer's point concerning the mature tRNA leves in the Northern blots and this is likely due to suboptimal hybridization conditions. We have adjusted the levels and contrast of the gel image in Fig. 5E so as to improve the visibility of the mature tRNA bands.

Furthermore, we believe that the intensity of the unspliced tRNA fragments is a sufficient readout of the perturbations of pre-tRNA splicing in our experiment. Assaying the levels of the mature forms was done in the complementary Leitner et al. manuscript so as to fully determine the extent of the defect caused by the FAM98 knock-outs. Moreover, the pre-tRNA splicing defect in FAM98B KO cells has also been recently reported in the Yang J et al. in Science (2025) paper.

In the present manuscript, the Northern blot data is only used to validate the structural findings by demonstrating that (i) there is a splicing defect in the FAM98B KO cells and that (ii) the defect can be rescued by overexpression of wild-type FAM98B but not by overexpressing a mutant protein that does not assemble within the tRNA-LC.

To reflect these points, we have adjusted the text in the Results section of the manuscript, where we now explicitly reference the complementary study by Leitner et al. (p. 9, lines 280-286):

"*As also shown in the complementary manuscript by Leitner et al., disruption of the endogenous FAM98A or FAM98C genes had no effect on tRNA^{Tyr} processing, suggesting that these paralogs are dispensable for tRNA ligation, while disruption of the FAM98B gene resulted in the accumulation of*

*unligated tRNA^{Tyr} fragments, indicating that FAM98B-containing tRNA-LC complex is required for efficient pre-tRNA splicing. The misprocessing phenotype was rescued by overexpression of wild-type FAM98B but not by expression of an FAM98B mutant incapable of tRNA-LC association (R221A/R228A/V229A/T232A/V233A), providing further validation for the structural role of FAM98B in tRNA-LC assembly (**Figure 5E**).*"
